# Increasing intratumor C/EBP-β LIP and nitric oxide levels overcome resistance to doxorubicin in triple negative breast cancer

**DOI:** 10.1186/s13046-018-0967-0

**Published:** 2018-11-27

**Authors:** Iris C. Salaroglio, Elena Gazzano, Ahmad Abdullrahman, Eleonora Mungo, Barbara Castella, Gamal Eldein Fathy Abd-ellatef Abd-elrahman, Massimo Massaia, Massimo Donadelli, Menachem Rubinstein, Chiara Riganti, Joanna Kopecka

**Affiliations:** 1 Department of Oncology, University of Torino, via Santena 5/bis, 10126 Turin, Italy; 2 Laboratory of Blood Tumor Immunology, Department of Molecular Biotechnology and Health Sciences, University of Torino, Turin, Italy; 3 Pharmaceutical and Drug Industries Research Division, Therapeutic Chemistry Department, National Research Centre, Cairo, Egypt; 4 Hematology Division, AO S Croce e Carle, Cuneo, Italy; 5 Department of Neurosciences, Biomedicine and Movement Sciences, University of Verona, Verona, Italy; 6 Department of Molecular Genetics, The Weizmann Institute of Science, Rehovot, Israel

**Keywords:** Triple negative breast cancer, Doxorubicin, P-glycoprotein, Endoplasmic reticulum stress, CAAT/enhancer binding protein (C/EBP)-β, Calreticulin

## Abstract

**Background:**

Triple negative breast cancer (TNBC) easily develops resistance to the first-line drug doxorubicin, because of the high levels of the drug efflux transporter P-glycoprotein (Pgp) and the activation of pro-survival pathways dependent on endoplasmic reticulum (ER). Interfering with these mechanisms may overcome the resistance to doxorubicin, a still unmet need in TNBC.

**Methods:**

We analyzed a panel of human and murine breast cancer cells for their resistance to doxorubicin, Pgp expression, lysosome and proteasome activity, nitrite production, ER-dependent cell death and immunogenic cell death parameters. We evaluated the efficacy of genetic (C/EBP-β LIP induction) and pharmacological strategies (lysosome and proteasome inhibitors), in restoring the ER-dependent and immunogenic-dependent cell death induced by doxorubicin, in vitro and in syngeneic mice bearing chemoresistant TNBC. The results were analyzed by one-way analysis of variance test.

**Results:**

We found that TNBC cells characterized by high levels of Pgp and resistance to doxorubicin, had low induction of the ER-dependent pro-apoptotic factor C/EBP-β LIP upon doxorubicin treatment and high activities of lysosome and proteasome that constitutively destroyed LIP. The combination of chloroquine and bortezomib restored doxorubicin sensitivity by activating multiple and interconnected mechanisms. First, chloroquine and bortezomib prevented C/EBP-β LIP degradation and activated LIP-dependent CHOP/TRB3/caspase 3 axis in response to doxorubicin. Second, C/EBP-β LIP down-regulated Pgp and up-regulated calreticulin that triggered the dendritic cell (DC)-mediated phagocytosis of tumor cell, followed by the activation of anti-tumor CD8^+^T-lymphocytes upon doxorubicin treatment. Third, chloroquine and bortezomib increased the endogenous production of nitric oxide that further induced C/EBP-β LIP and inhibited Pgp activity, enhancing doxorubicin’s cytotoxicity. In orthotopic models of resistant TNBC, intratumor C/EBP-β LIP induction - achieved by a specific expression vector or by chloroquine and bortezomib - effectively reduced tumor growth and Pgp expression, increased intra-tumor apoptosis and anti-tumor immune-infiltrate, rescuing the efficacy of doxorubicin.

**Conclusions:**

We suggest that preventing C/EBP-β LIP degradation by lysosome and proteasome inhibitors triggers multiple virtuous circuitries that restore ER-dependent apoptosis, down-regulate Pgp and re-activate the DC/CD8^+^T-lymphocytes response against TNBC. Lysosome and proteasome inhibitors associated with doxorubicin may overcome the resistance to the drug in TNBC.

**Electronic supplementary material:**

The online version of this article (10.1186/s13046-018-0967-0) contains supplementary material, which is available to authorized users.

## Background

Triple negative breast cancer (TNBC) is often treated with anthracycline (e.g. doxorubicin or daunorubicin)- or taxane-based monotherapy [1], but the success is lower than in other breast cancer types [2].

Doxorubicin kills tumor cells by inducing DNA damage, increasing reactive oxygen and nitrogen species such as nitric oxide (NO), impairing mitochondrial metabolism, inducing endoplasmic reticulum (ER) stress and immunogenic cell death (ICD) [3–5]. The main mechanism of doxorubicin-induced ICD is the induction of ER stress, that triggers the translocation of calreticulin (CRT) from the ER, where it works as calcium sensor and chaperon, to the plasma-membrane. Here, CRT promotes the phagocytosis of tumor cells by dendritic cells (DC) and the activation of a durable anti-tumor response by CD8^+^T-lymphocytes [6].

Doxorubicin’s efficacy is limited by the presence of drug efflux transporters such as P-glycoprotein (Pgp) [7]. Pgp limits the doxorubicin intracellular accumulation and the drug’s ability to elicit pleiotropic cytotoxic effects.

Pgp expression is regulated by multiple transcription factors. CAAT/enhancer binding protein (C/EBP)-β, a transcription factor with two isoforms - C/EBP-β LAP and LIP - that work as antagonists, is one of the main controller of Pgp expression in solid tumors [5]. LAP is activated during early ER stress, induces pro-survival pathways and up-regulates Pgp; LIP is induced after prolonged ER stress, stimulates C/EBP homologous protein (CHOP)/Tribbles 3(TRB3)/caspase 3-mediated apoptosis [8] and down-regulates Pgp [9].

Besides a high expression, also a high activity of Pgp determines doxorubicin resistance. Natural and synthetic inhibitors of Pgp [10, 11], liposomal formulations [12], co-delivery of Pgp inhibitors plus doxorubicin [13], have been tested to reduce Pgp activity in vitro and in preclinical models, but until now none of these approaches were effective in patients. NO is a potent inhibitor of Pgp activity: this molecule, released by synthetic NO donors or produced by the endogenous NO synthase (NOS) enzymes, nitrates specific tyrosines that are critical for Pgp activity. Such covalent modification reduces the doxorubicin efflux through Pgp [14–16]. Curiously, doxorubicin increases the endogenous production of NO, that mediates part of the cytotoxic effects of the drug [16], stimulates the translocation of CRT and the ICD of tumor cell [17], induces ER stress [18, 19]. These events, however, occur only in doxorubicin-sensitive/Pgp-negative cells, not in doxorubicin-resistant/Pgp-positive ones [16, 17], leading to hypothesize that multiple cross-talks determine a chemo-immune-resistant phenotype. Indeed, Pgp-positive cancer cells: i) do not accumulate the intracellular amount of doxorubicin sufficient to increase NO production [16] and induce ICD [17]; ii) do not induce C/EBP-β LIP and ER stress-dependent cell death [5], a condition necessary for the translocation of CRT on cell surface and the subsequent ICD [3]; iii) are not phagocytized by DC since Pgp hampers the immune-activating functions of CRT in plasma-membrane [20].

Disrupting these vicious circles by decreasing Pgp expression and activity is the only way to restore the multiple cytotoxic mechanisms of doxorubicin. In this work we demonstrated that preventing C/EBP-β LIP degradation and increasing NO levels reduce at the same time expression and activity of Pgp, restore the ER stress-dependent apoptosis and the ICD induced by doxorubicin, rescuing the anthracycline’s therapeutic efficacy in Pgp-positive TNBC.

## Materials and methods

### Chemicals and supplies

Plastic ware were obtained from Falcon (Becton Dickinson, Franklin Lakes, NJ). Electrophoresis reagents were from Bio-Rad Laboratories (Hercules, CA). The protein content of cell lysates was assessed using the BCA kit from Sigma Chemicals Co. (St. Louis, MO). Unless specified otherwise, all reagents were purchased from Sigma Chemicals Co.

### Cells

Human non-transformed breast epithelial MCF10A cells, human breast cancer MCF7, SKBR3, T47D, MDA-MB-231 cells, murine mammary cancer JC cells were purchased from ATCC (Manassas, VA). Murine mammary cancer TUBO cells were a kind gift of Prof. Federica Cavallo, Department of Molecular Biotechnology and Health Sciences, University of Torino, Italy. All human cells were authenticated by microsatellite analysis using the PowerPlex kit (Promega Corporation, Madison, WI; last authentication: January 2018). For 3D-cultures, 1 × 10^5^ cells were seeded in 96-well plate coated with Biomimesys™ matrix (Celenys, Rouen, France). Cells were grown in DMEM/HAM F12 nutrient mixture medium (MCF710A, MCF7, SKBR3, T47D), RPMI-1640 medium (MDA-MB-231, JC), DMEM medium (TUBO) supplemented with 10% *v*/v fetal bovine serum (FBS) and 1% v/v penicillin-streptomycin. Cells were checked for *Mycoplasma spp.* contamination by PCR every three weeks; contaminated cells were discharged.

### Immunoblotting

Plasma-membrane proteins were isolated using the Cell Surface Protein Isolation kit (ThermoFisher Scientific Inc., Waltham, MA) according to the manufacturer’s protocol. For whole cell lysates, cells were rinsed with lysis buffer (50 mM Tris-HCl, 1 mM EDTA, 1 mM EGTA, 150 mM NaCl, 1% v/v Triton-X100; pH 7.4), supplemented with the protease inhibitor cocktail III (Cabiochem, La Jolla, CA), sonicated and clarified at 13000×g, for 10 min at 4 °C. Protein extracts (20 μg) were subjected to SDS-PAGE and probed with the following antibodies: anti-Pgp (1:250, rabbit polyclonal, #sc-8313, Santa Cruz Biotechnology Inc., Santa Cruz, CA), anti-multidrug resistant protein 1 (MRP1; 1:500, mouse clone MRPm5, Abcam, Cambridge, UK), anti-breast cancer resistance protein (1:500, mouse clone BXP-21, Santa Cruz Biotechnology Inc.), anti-C/EBP-β (1:500, rabbit polyclonal, # sc150, Santa Cruz Biotechnology Inc.), anti-CHOP (1:500, mouse monoclonal, #ab11419, Abcam), anti-TRB3 (1:500, rabbit polyclonal, #13300–1-AP, Proteintech, Chicago, IL), anti-caspase-3 (1:1000, mouse clone C33, GeneTex, Hsinhu City, Taiwan), anti-CRT (rabbit polyclonal #PA3–900, Affinity Bioreagents, Rockford, IL), anti-NOS I (1:500, mouse clone 16, BD Biosciences, Franklin Lakes, NJ), anti-NOS II (1:1000, mouse clone 4E5, ThermoFisher Scientific Inc.), anti-NOS III (1:500, mouse clone 3, BD Biosciences), anti-pancadherin (1:500, goat clone C-19, Santa Cruz Biotechnology Inc.), anti-β-tubulin (1:1000, mouse clone D10, Santa Cruz Biotechnology Inc.), followed by the horseradish peroxidase-conjugated secondary antibodies (Bio-Rad). The membranes were washed with Tris-buffered saline (TBS)/Tween 0.01% *v*/v. To detect ubiquitinated C/EBP-β, 100 μg of proteins from whole cell lysates were immuno-precipitated overnight with the anti-C/EBP-β antibody, then probed with an anti-mono/poly-ubiquitin antibody (1:1000, mouse clone FK2, Axxora, Lausanne, Switzerland), using 50 μl of PureProteome Magnetic Beads (Millipore, Bedford, MA). To detect nitrated Pgp, 100 μg of proteins from plasma-membrane were immuno-precipitated overnight with anti-nitrotyrosine antibody (1:50, rabbit polyclonal, #06–284, Millipore), then probed with the anti-Pgp antibody. Proteins were detected by enhanced chemiluminescence (Bio-Rad Laboratories). Blot images were acquired with a ChemiDocTM Touch Imaging System device (Bio-Rad Laboratories). The densitometric analysis was performed with the ImageJ software (https://imagej.nih.gov/ij).

### Lysosome and proteasome activities

The activity of cathepsin L, an index of lysosome activity, was measured according to [21]. The results were expressed as nmoles/mg cellular proteins. Proteasome activity was measured with the Proteasome-Glo™ Cell-Based Assays (Promega Corporation). The results were expressed as relative luminescence units (RLU)/mg cellular proteins.

### Cell viability

1 × 10^4^ cells were seeded in 96-well plate and incubated as described in the experimental section for 72 h. To calculate the IC_50_, cells were treated with doxorubicin at scalar concentrations (from 10^− 10^ to 10^− 3^ M). Viability was measured with the ATPLite Luminescence Assay kit (PerkinElmer, Waltham, MA) as per manufacturer’s instructions. The viability in untreated cells was considered as 100%. The results were expressed as percentage of viable cells towards the untreated cells. The IC_50_ was calculated with the CompuSyn software (http://www.combosyn.com).

### Doxorubicin accumulation and efflux

The intracellular doxorubicin content and the drug efflux were measured as detailed in [16]. The intracellular doxorubicin concentration was expressed as nanomoles of doxorubicin/mg cellular proteins. The efflux of doxorubicin was expressed as the change in the intracellular concentration of the drug/minute (dc/dt).

### Pgp ATPase activity

The Pgp ATPase activity was measured in Pgp-rich membrane vesicles as described in [22]. The results were expressed as μmol hydrolyzed phosphate/min/mg membrane proteins.

### Nitrite production and NOS activity

The production of nitrite, the stable derivative of NO, was measured spectrophotometrically by the Griess methods, as described in [23]. Nitrite concentration was expressed as nanomoles/min/mg cellular proteins. The activity of NOS in cell lysates was measured using the Ultrasensitive Colorimetric Assay for Nitric Oxide Synthase kit (Oxford Biomedical Research, Oxford, MI), as per manufacturer’s instructions. The enzyme activity was expressed as nanomoles of nitrites/min/mg cellular proteins.

### Immunofluorescence analysis

5 × 10^5^ 2D-cells were grown onto glass coverslips in 24-well plates overnight; the same number of cells was seed to produce 3D-cultures, analyzed after 1 week. Sample were fixed using 4% *w*/*v* paraformaldehyde (PFA) for 15 min at room temperature, washed with PBS, incubated for 1 h at 4 °C with an anti-Pgp antibody (1:50, mouse clone JSB-1; Abcam, diluted in 1% FBS/PBS), washed five times with PBS and incubated for 1 h at room temperature with an AlexaFluor488-conjugated secondary antibody (Abcam, diluted 1:50 in 1% FBS/PBS). Cells were incubated with 4′,6-diamidino-2-phenylindole dihydrochloride (DAPI), diluted 1:10000 in PBS for 5 min, washed four times with PBS and once with deionized water. The cover slips were mounted with the Gel Mount Aqueous Mounting and examined with a Leica DC100 fluorescence microscope (Leica Microsystems GmbH, Wetzlar, Germany). For each experimental point, a minimum of five microscopic fields were examined.

### Over-expression of C/EBP-β LAP and LIP

The pcDNA4/TO expression vectors (Invitrogen Life Technologies, Milan, Italy) for LAP and LIP, produced as reported previously [8], were co-transduced with pcDNA6/TR vector (Invitrogen Life Technologies) in parental cells. Doxycycline-inducible (TetON) stable clones were generated by selecting cells with 2 μg/ml blasticidin S (Invitrogen Life Technologies) and 100 μg/ml zeocin (InvivoGen, San Diego, CA). LIP induction was activated by adding 1 μg/ml doxycycline in the culture medium.

### Quantitative real time-PCR (qRT-PCR)

Total RNA was extracted and reverse-transcribed using the iScriptTM cDNA Synthesis Kit (Bio-Rad Laboratories). qRT-PCR was performed using the IQ™ SYBR Green Supermix (Bio-Rad Laboratories). The following PCR primer sequences were designed using the qPrimerDepot software (http://primerdepot.nci.nih.gov/): *Pgp* (human): 5’-TGCTGGAGCGGTTCTACG*-*3′, 5’*-*ATAGGCAATGTTCTCAGCAATG-3′; *Pgp* (mouse): 5’-TGCTTATGGATCCCAGAGTGAC*-*3′, 5’*-*TTGGTGAGGATCTCTCCGGCT-3’;*CRT* (human): 5’-TGTCAAAGATGGTGCCAGAC-3′, 5’-ACAACCCCGAGTATTCTCCC-3′; *CRT* (mouse): 5’-TACAAGGGCGAGTGGAAACC-3′, 5’-GCATCGGGGGAGTATTCAGG-3′; *S14* (human): 5’-CGAGGCTGATGACCTGTTCT-3′, 5’-GCCCTCTCCCACTCTCTCTT-3′; *S14* (mouse): 5’-TCTGGGATGAAGATTGGGCG-3′, 5’-ACCCCCTTTTCTTCGAGTGC-3′. The relative gene expression levels were calculated using the Gene Expression Quantitation software (Bio-Rad Laboratories).

### Chromatin immunoprecipitation (ChIP)

The putative binding sites of C/EBP-β, containing CAAT box motif, on human and murine *CRT* promoter were identified using the Gene Promoter Miner software (http://gpminer.mbc.nctu.edu.tw/). The following primers were designed with the Primer3 software (http://primer3.ut.ee/): 5′-TGGGGAGGTGGAGTAGAGTG-3′; 5’-CAGGAACTGCAGGGACTGAG-3′ (site 831–843, human *CRT* promoter); 5’-CTCACAGGTCTCGCCTTGTC-3′; 5’-ATGCACTGTTCCGACGTTC-3′ (site 1302–1313, human *CRT* promoter); 5’-CCTAGCGAGCCAGAGACTC-3′; 5’-CTATTGGTCGCACTATGGGC-3′ (site 798–811, mouse *CRT* promoter); 5’-GCCTAACTTGCTGAGCCAAC-3′; 5’-CTACCTCTCACCCGAACCTG-3′ (site 872–883, mouse *CRT* promoter). To determine the binding of LAP and LIP to *CRT* promoter, ChIP was performed as described in [24].

### Flow cytometry analysis

1 × 10^5^ cells were washed with PBS, detached with Cell Dissociation Solution, wash twice with PBS, incubated for 45 min at 4 °C with the anti-CRT antibody, diluted 1:100 in 0.25% *v*/*v* bovine serum albumin (BSA)-PBS, followed by the AlexaFluor488-conjugated secondary antibody (1:50) for 30 min at 4 °C. After the fixation step in 2.5% *v*/v PFA for 5 min at room temperature, samples were analyzed with a Guava*®* EasyCyte flow cytometer (Millipore) equipped with the InCyte software (Millipore). Cells incubated with not-immune isotype antibody, followed by secondary antibody, were include as control of specificity.

### Tumor cells phagocytosis and T-lymphocytes activation

DC were generated from monocytes immuno-magnetically isolated from peripheral blood of healthy donors, provided by Blood Bank of AOU Città della Salute e della Scienza, Torino, Italy as previously reported [25] or from the bone marrow of 6-week old female balb/C mice [26]. The phagocytosis assay was performed as detailed in [26], by co-incubating DC and tumor cells at 37 °C and 4 °C for 24 h. The percentage of phagocytized cells obtained after the incubation at 4 °C was subtracted from the percentage obtained at 37 °C, and was always less than 5% (not shown). The phagocytosis rate was expressed as phagocytic index [26]. After cell phagocytosis, DC were washed and co-cultured for 10 days with autologous T-cells, isolated by immuno-magnetic sorting with the Pan T Cell Isolation Kit (Miltenyi Biotec., Tetrow, Germany). The expression of CD107, a degranulation marker and an index of active cytotoxic CD8^+^T-lymphocytes, was determined by flow cytometry as previously reported [25], using anti-human or mouse fluorescein isothyocyanate (FITC)-conjugated-CD8 (1:10, clones BW135/80 and 53–6.7) and phycoerythrin (PE)-conjugated-CD107 (1:10, clones H4A3 and 1D4B) antibodies (Miltenyi Biotec).

### Cell silencing

1 × 10^5^ cells were treated with 10 nM of 3 unique 27mer siRNA duplexes, targeting DDIT3/CHOP (#SR319903; Origene, Rockville, MD) or with a Trilencer-27 Universal scrambled negative control siRNA duplex (#SR30004; Origene), as per manufacturer’s instructions. The efficiency of silencing was verified by immunoblotting.

### Calreticulin knock-out (KO)

JC cells were knocked-out for calreticulin using a pool of two calreticulin-targeting CRISPR/Cas9 KO-green fluorescence protein (GFP) vectors (#KN302469, Origene). Non-targeting (scrambled) CRISPR/Cas9 vector (Origene) was used as control of specificity. 1 × 10^5^ cells were seeded in antibiotic-free medium. 1 μg of CRISPR/Cas9 plasmid was used, as per manufacturer’s instructions. Transfected cells were sorted by isolating GFP-positive cells. KO efficacy was verified by immunoblotting. Stable KO-clones were generated by culturing cells for 6 weeks in medium containing 1 μg/ml puromycin.

### In vivo tumor growth

1 × 10^7^ JC TetON LIP cells, wild-type, stably transfected with a KO-CRT vector or with a scrambled vector, were mixed with 100 μl Matrigel and orthotopically implanted in 6 week-old female immunocompetent balb/C mice (Charles River Laboratories Italia, Calco), housed (5 per cage) under 12 h light/dark cycle, with food and drinking provided ad libitum. Tumor growth was measured daily by caliper, according to the equation (LxW^2^)/2, where L = tumor length and W = tumor width. When tumor reached the volume of 50 mm^3^, mice were randomized and treated as reported in the experimental section. Tumor volumes were monitored daily. Animals were euthanized at day 21 after randomization with zolazepam (0.2 ml/kg) and xylazine (16 mg/kg). Lactate dehydrogenase, aspartate aminotransferase, alanine aminotransferase, alkaline phosphatase, creatinine, creatine phosphokinase and troponin were measured on blood samples collected immediately after euthanasia, using commercially available kits from Beckman Coulter Inc. (Beckman Coulter, Miami, FL). In all studies, researchers analyzing the results were unaware of the treatments received by animals.

### Immunohistochemistry analysis

Tumors were resected and fixed in 4% *v*/v PFA, photographed and sectioned, then stained with hematoxylin/eosin or immuno-stained for Ki67 (1:50, rabbit polyclonal #AB9260, Millipore), Pgp (1:50), CHOP (1:50), cleaved(Asp175)-caspase 3 (1:200, rabbit polyclonal #9661, Cell Signaling Technology Inc., Danvers, MA), CRT (1:100), CD11c (1:50, hamster, clone HL3, BD Biosciences) to label intra-tumor DC, CD8 (1:100, rat clone YTS169.4, Abcam) to label intra-tumor cytotoxic T-lymphocytes, followed by a peroxidase-conjugated secondary antibody (1:100, Dako, Glostrup, Denmark). Sections were examined with a Leica DC100 microscope.

### IFN-γ production

Tumor-draining lymph nodes were collected, homogenized for 30 s at 15 Hz using a TissueLyser II device (Qiagen, Hilden, Germany) and centrifuged at 12000×g for 5 min. The supernatant was collected to measure the amount of IFN-γ, using the Mouse IFN-γ DuoSet ELISA Kit (R&D Systems, Minneapolis, MN). The results were expressed as nmol/ml.

### Statistical analysis

All data in the text and figures are provided as means±SD. The results were analysed by a one-way analysis of variance (ANOVA), using the Statistical Package for Social Science (SPSS) software (IBM, Armonk, NY). *p* < 0.05 was considered significant.

## Results

### Pgp-positive breast cancer cells do not induce C/EBP-β LIP upon doxorubicin treatment and have high lysosome and proteasome activities

**Fig. 1:**
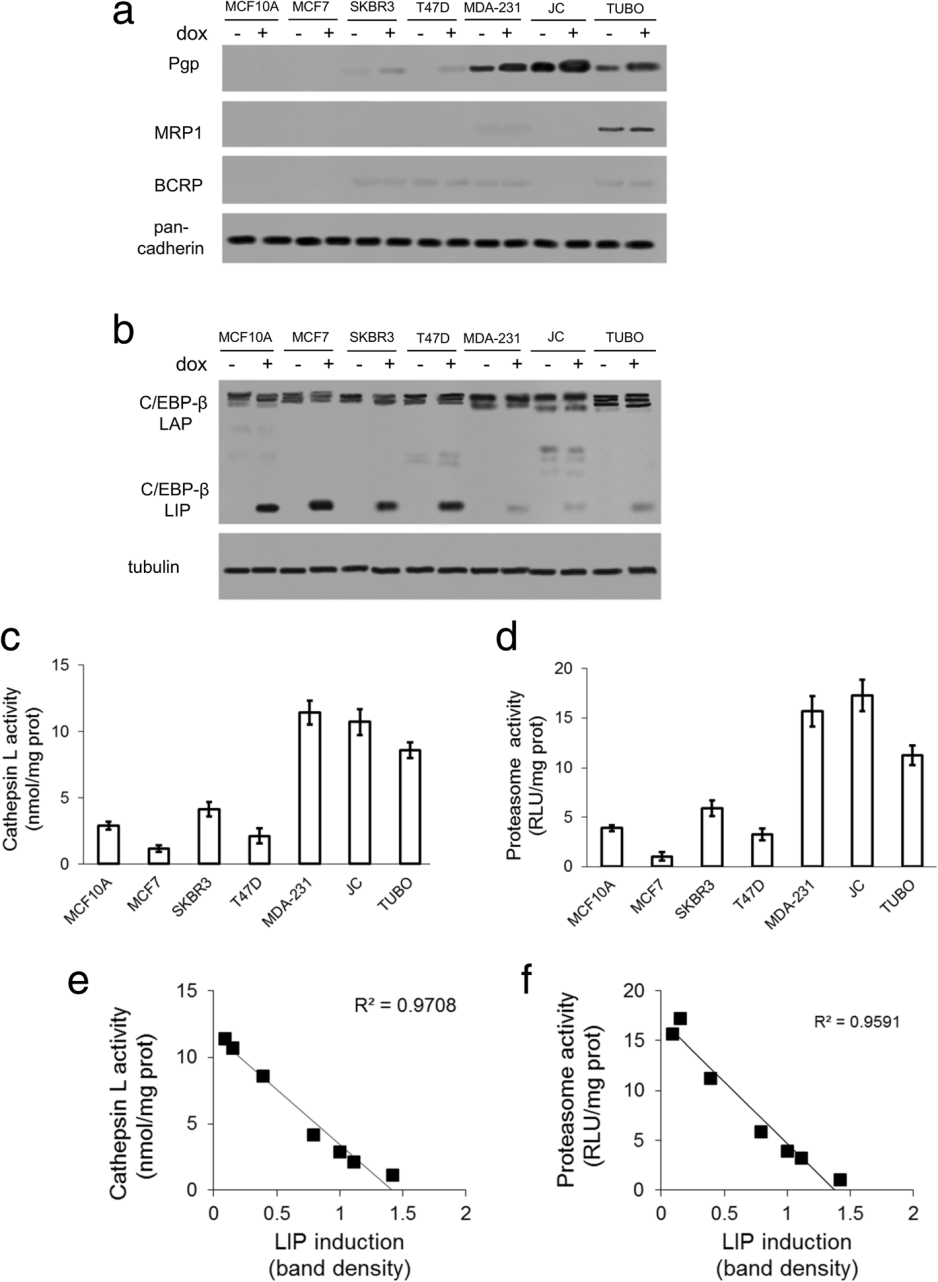
Doxorubicin induces C/EBP-β LIP in Pgp-negative but not in Pgp-positive breast cancer cells. Cells were cultured in the absence (−) or presence (+) of 5 μM doxorubicin (dox) for 24 h. **a**. Plasma-membrane extracts were probed with the indicated antibodies. The expression of pancadherin was used as control of equal protein loading. The figure is representative of 1 out of 3 experiments. **b**. Whole cell lysates were probed with an antibody recognizing both C/EBP-β LAP and LIP isoforms. The expression of β-tubulin was used as control of equal protein loading. The figure is representative of 1 out of 3 experiments. **c**-**d**. Lysosome activity (panel **c**) was analyzed in duplicates by a spectrophotometric assay, proteasome activity (panel **d**) was analyzed in duplicates by a chemiluminescence-based assay. Data are presented as means±SD (*n* = 3). **e-f.** Correlation between LIP band density upon doxorubicin treatment (panel **b**) and lysosome (panel **c**) or proteasome (panel **d**) activities. The mean band density of LIP was expressed as arbitrary units using the ImageJ software, setting the mean band density in MCF10A cells as 1

The analysis of ABC transporters (Pgp, MRP1, BCRP) involved in doxorubicin efflux in epithelial mammary MCF10A cells and in a panel of breast cancer cell lines indicated a higher expression of Pgp in TNBC human MDA-MB-231cells, and in murine JC and TUBO cells. Pgp levels were further increased by doxorubicin exposure. None cell line expressed MRP1, except TUBO cells, without changes induced by doxorubicin. BCRP levels were undetectable or very low (Fig. 1a). The pattern of Pgp expression was in accord with the IC_50_ to doxorubicin of each cell line (Additional file 1). Either untreated or doxorubicin-treated cells expressed C/EBP-β LAP. C/EBP-β LIP was induced by doxorubicin in epithelial MCF10A cells and in Pgp-negative breast cancer MCF7, SKBR3, and T47D cells, while Pgp-positive MDA-MB-231, JC and TUBO cells had a lower C/EBP-β LIP induction (Fig. 1b). These three cell lines had higher lysosome (Fig. 1c) and proteasome (Fig. 1d) activities compared to doxorubicin-sensitive/Pgp-negative cells. The amount of LIP induced upon doxorubicin treatment was inversely correlated with the lysosome (Fig. 1e) and proteasome (Fig. 1f) activity in the cell lines examined, suggesting that – as observed for other solid tumors [5, 27] – the degradation via lysosome and proteasome is critical in preserving C/EBP-β LIP level in breast cancer.

### Doxorubicin resistance is associated to the lack of C/EBP LIP-β-dependent apoptosis in breast cancer cells

We thus examined if the FDA-approved lysosome inhibitor chloroquine and proteasome inhibitor bortezomib could prevent C/EBP-β LIP degradation. In preliminary dose-dependence experiments, we determined that at 1 μM chloroquine and bortezomib - used as single agents - did not reduce significantly cell viability (Additional file 2), but they decreased lysosome (Additional file 3a) and proteasome (Additional file 3b) activities, respectively, in Pgp-positive MDA-MB-231 and in JC cells. When used in combination, chloroquine and bortezomib significantly decreased the viability of these cell lines (Additional file 2).

**Fig. 2:**
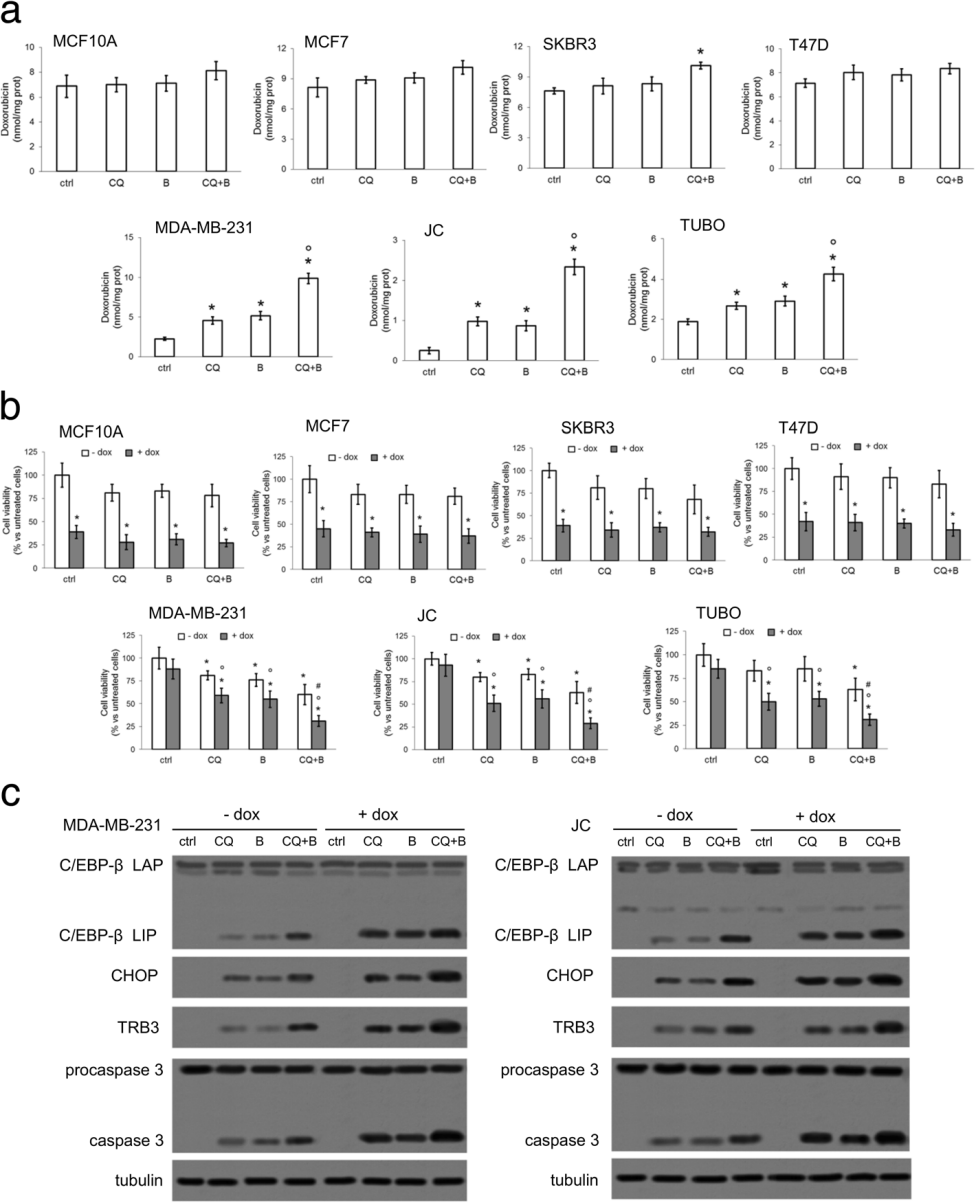
Chloroquine and bortezomib restore doxorubicin accumulation, cell death and C/EBP-β LIP induction in Pgp-positive cells. Cells were cultured in the absence (ctrl) or presence of the lysosome inhibitor chloroquine (CQ; 1 μM), the proteasome inhibitor bortezomib (B; 1 μM), or their combination, for 24 h (panels **a**-**c**) or 72 h (panel **b**). Doxorubicin (dox; 5 μM) was added for additional 24 h (panels **a**-**c**) or in the last 48 h (panel **b**). **a.** Doxorubicin accumulation was measured in triplicates by a fluorimetric assay. Data are presented as means±SD (*n* = 3). *p < 0.01: treated cells vs ctrl cells; °*p* < 0.001: CQ + B-treated cells vs CQ/B-treated cells. **b.** Cell viability was measured in quadruplicates by a chemiluminescence-based assay. Data are presented as means±SD (n = 3). *p < 0.02: treated cells vs “- dox ctrl” cells; °*p* < 0.02: treated cells vs “+ dox ctrl” cells; ^#^*p* < 0.005: CQ + B-treated cells vs CQ/B-treated cells. **c.** Whole cell lysates were probed with the indicated antibodies. The expression of β-tubulin was used as control of equal protein loading. The figure is representative of 1 out of 3 experiments

In Pgp-negative MCF10A, MCF7, SKBR3 or T47D cells, which accumulated higher amount of doxorubicin compared to MDA-MB-231, JC or TUBO cells, the combination of chloroquine and bortezomib did not increase the drug content (Fig. 2a) nor enhanced the anti-proliferative effects of doxorubicin **(**Fig. 2b). By contrast, chloroquine and bortezomib, in particular when used in combination, significantly increased doxorubicin accumulation (Fig. 2a) and cytotoxicity (Fig. 2b) in Pgp-positive MDA-MB-231, JC and TUBO cells. For the following investigations, we focused on the TNBC human MDA-MB-231 cells and on murine JC cells, i.e. the cell lines characterized by the highest expression of Pgp, the lowest induction of C/EBP-β LIP, the highest lysosome and proteasome activities (Fig. 1a-c). In both cell lines, chloroquine and bortezomib increased C/EBP-β LIP and the LIP-dependent CHOP/TRB3/caspase 3 axis, while doxorubicin did not. The induction of C/EBP-β LIP/CHOP/TRB3/caspase 3 axis was stronger in cells treated with chloroquine and bortezomib followed by doxorubicin (Fig. 2c). These results suggest that chloroquine, bortezomib and doxorubicin cooperate each other in increasing C/EBP-β LIP and the downstream pro-apoptotic CHOP/TRB3/caspase 3 axis.

### Chloroquine and bortezomib down-regulate Pgp expression and activity by increasing C/EBP-β LIP and NO

**Fig. 3:**
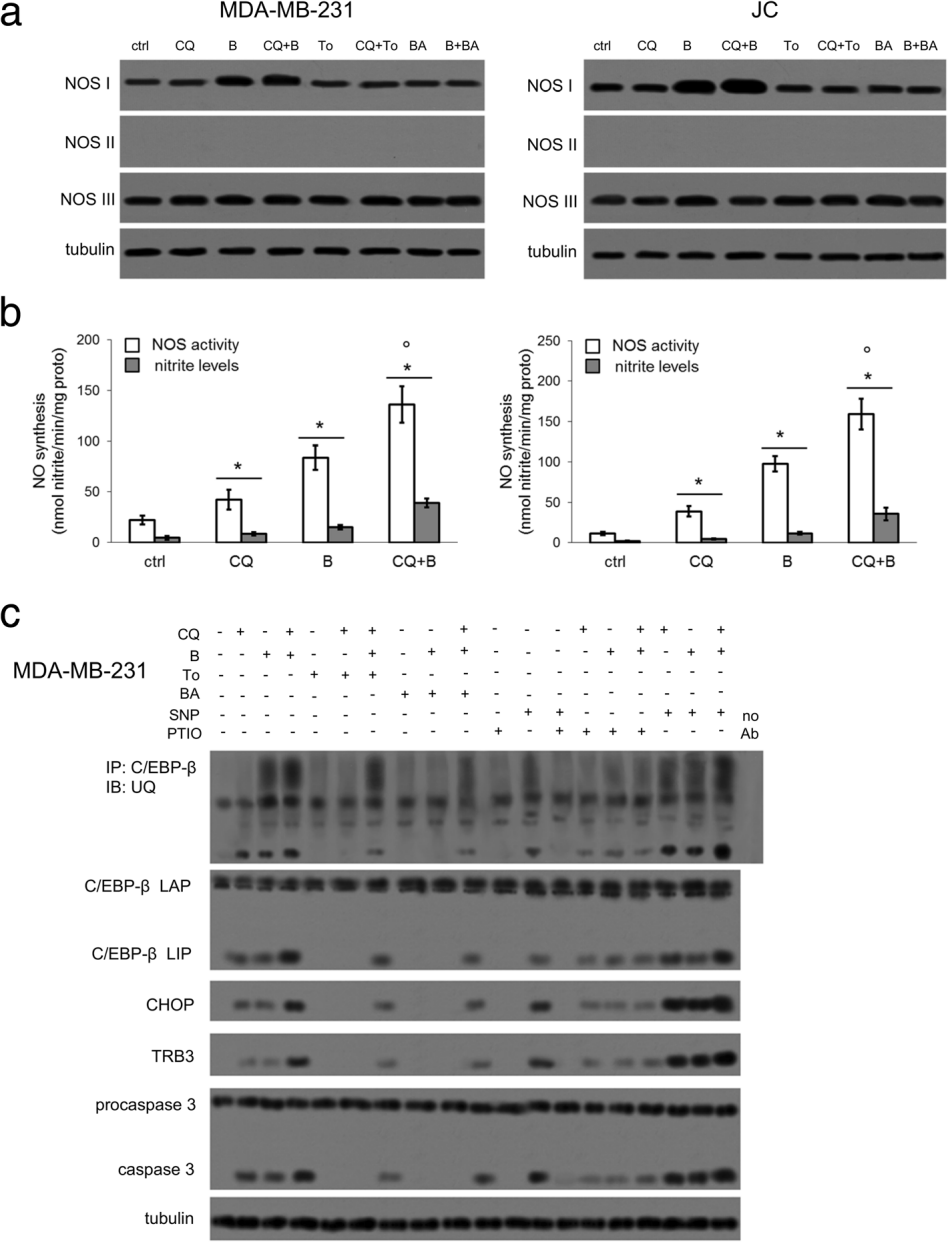
Chloroquine and bortezomib induce C/EBP-β LIP/CHOP/TRB3/caspase 3 axis by increasing NO levels. MDA-MB-231 and JC cells were cultured for 24 h in the absence (ctrl, −) or presence (+) of the lysosome inhibitor chloroquine (CQ; 1 μM) or of the proteasome inhibitor bortezomib (B; 1 μM), alone or in combination. When indicated, the lysosome activator torin-1 (To; 1 μM) or the proteasome activator betulinic acid (BA; 10 μM) were added. **a.** Whole cell lysates were probed for NOS I, NOS II, NOS III. The expression of β-tubulin was used as control of equal protein loading. The figure is representative of 1 out of 3 experiments. **b.** The activity of NOS enzyme in cell lysate and the levels of nitrite in the supernatants were measured in triplicates by spectrophotometric assays. Data are presented as means±SD (*n* = 3). *p < 0.05: treated cells vs ctrl cells; °*p* < 0.02: CQ + B-treated cells vs CQ/B-treated cells. **c.** MDA-MB-231 cells were cultured for 24 h in the absence (−) or presence (+) of the lysosome inhibitor chloroquine (CQ; 1 μM), the proteasome inhibitor bortezomib (B; 1 μM), the lysosome activator torin-1 (To; 1 μM), the proteasome activator betulinic acid (BA; 10 μM), the NO donor sodium nitroprusside (SNP; 10 μM), the NO scavenger carboxy-PTIO (PTIO; 100 μM), alone or co-incubated in different combinations. Whole cell lysates were immunoprecipitated (IP) with the anti-C/EBP-β antibody, which recognizes both C/EBP-β-LAP and C/EBP-β-LIP, then immunoblotted (IB) with the anti-mono/poly-ubiquitin (UQ) antibody; alternatively, lysates were directly immunoblotted with the indicated antibodies. No Ab: lysate from untreated cells immunoprecipitated in the absence of the anti-C/EBP-β antibody, as control of specificity. The expression of β-tubulin was used as control of equal protein loading before immunoprecipitation. The figure is representative of 1 out of 3 experiments

NOS I and NOS III constitutively produce NO, while NOS II is induced upon inflammation [28] or doxorubicin in drug-sensitive cells [16]. MDA-MB-231 and JC cells basally express NOS I and NOS III, while NOS II was undetectable (Fig. 3a).

It has been previously reported that NOS I expression is increased by the inhibition of proteasome [29], while NOS III activity is increased by chloroquine [30]: indeed, the lysosome inhibitor lowers the availability of intracellular free iron [31]; this condition increases NOS III activity without changing its expression [30]. In line with these findings, bortezomib, alone or in combination with chloroquine, increased NOS I expression, while chloroquine did not alter the expression of any NOS isoform (Fig. 3a). The use of the proteasome activator betulinic acid suggested that the up-regulation in NOS I induced by bortezomib was mediated by the inhibition of proteasome activity (Fig. 3a). Both chloroquine and bortezomib, alone and in particular in combination, increased the activity of NOS enzymes and the production of nitrite, the stable derivative of NO (Fig. 3b; Additional file 4). This trend can be due either to the increased expression of NOS I induced by bortezomib (Fig. 3a) or to the increased activity of NOS III induced by chloroquine [30].

Since NO induces ER stress [19], we performed the following add-back experiments in order to investigate whether chloroquine and bortezomib up-regulated C/EBP-β LIP by inhibiting lysosomal and proteasomal activity, by increasing NO production or by both mechanisms.

In a first experimental set, we co-incubated chloroquine and bortezomib with the lysosome activator torin-1 or the proteasome activator betulinic acid. As shown in Fig. 3c and Additional file 5, in both MDA-MB-231 and JC cells, torin-1 reduced the induction of C/EBP-β LIP/CHOP/TRB3/caspase 3 axis elicited by chloroquine, alone or combined with bortezomib. Since torin-1 did not affect the ubiquitination of C/EBP-β LIP, its effect was likely due to the activation of the LIP degradation via lysosome. Betulinic acid reduced the up-regulation of C/EBP-β LIP and downstream effectors induced by bortezomib, alone or associated with chloroquine. Of note, betulinic acid also reduced the levels of poly-ubiquitinated LIP, indicating that if favored the removal of ubiquitinated LIP via proteasome.

In a second experimental set, we used the NO donor sodium nitroprusside (SNP), which increased nitrite, and the NO scavenger carboxy-PTIO, which reduced the amount of nitrite in untreated, chloroquine- and bortezomib-treated cells (Additional file 4). SNP increased C/EBP-β LIP levels and CHOP/TRB3/caspase 3 axis activation, while the co-incubation with carboxy-PTIO abrogated these events (Fig. 3c; Additional file 5), suggesting the increased levels of NO may trigger the induction of LIP and ER-dependent apoptotic cascade. Of note, PTIO also reduced the increase in nitrite (Additional file 4) and in C/EBP-β LIP/CHOP/TRB3/caspase 3 axis in cells co-incubated with chloroquine and bortezomib (Fig. 3c; Additional file 5). We did not find a further enhancement of nitrite or C/EBP-β LIP/CHOP/TRB3/caspase 3 up-regulation in cells treated with SNP, chloroquine and/or bortezomib compared to cells treated with SNP alone (Fig. 3c; Additional files 4, 5), suggesting that in these experimental conditions the level of NO released by SNP was likely saturating and sufficient to reach the maximal C/EBP-β LIP induction.

Overall these results suggest that either the inhibition of lysosome and proteasome activity or the increase in endogenous NO mediate the induction of C/EBP-β LIP exerted by chloroquine and bortezomib.

**Fig. 4:**
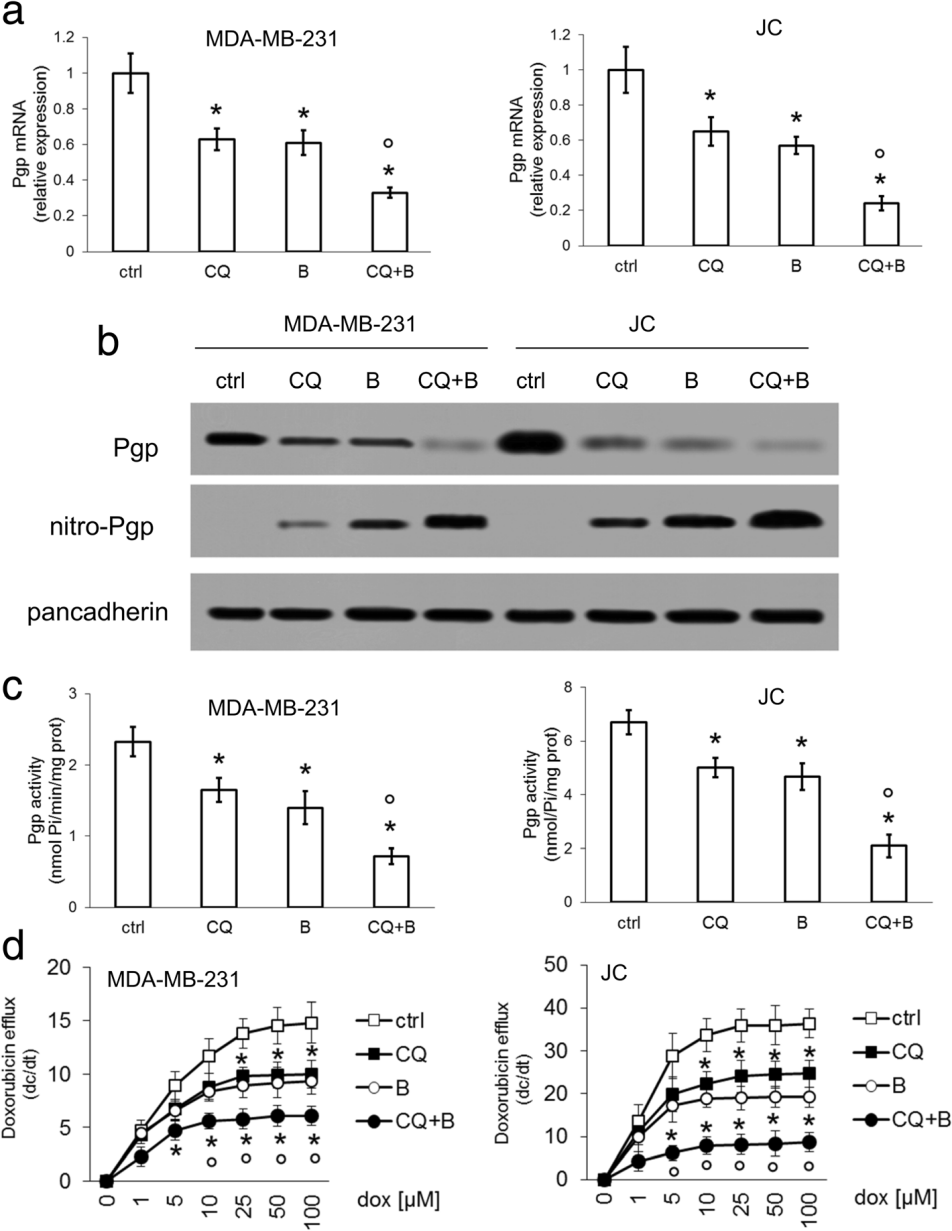
Chloroquine and bortezomib downregulate expression and activity of Pgp. MDA-MB-231 and JC cells were cultured for 24 h in the absence (ctrl) or presence of the lysosome inhibitor chloroquine (CQ; 1 μM) or the proteasome inhibitor bortezomib (B; 1 μM), alone or in combination. **a.** The relative expression of *Pgp* gene was measured by qRT-PCR. Data are presented as means±SD (*n* = 4). *p < 0.02: treated cells vs ctrl cells; °*p* < 0.001: CQ + B-treated cells vs CQ/B-treated cells. **b.** Plasma-membrane extracts were probed for Pgp or immunoprecipitated with an anti-nitrotyrosine antibody, then probed for Pgp (nitroPgp). The expression of pancadherin was used as control of equal membrane protein loading. The figure is representative of 1 out of 3 experiments. **c.** Pgp activity was analyzed in duplicates by a spectrophotometric assay. Data are presented as means±SD (n = 4). *p < 0.02: treated cells vs ctrl cells; °*p* < 0.002: CQ + B-treated cells vs CQ/B-treated cells. **d.** Doxorubicin efflux (i.e. the change of intracellular doxorubicin concentration per unit of time; dc/dt) was measured in triplicates by a fluorimetric assay, in cells incubated 10 min with increasing concentrations of doxorubicin to achieve the maximal velocity efflux (Vmax). Data are presented as means±SD (*n* = 3). *p < 0.001: treated cells vs ctrl cells; °*p* < 0.001: CQ + B-treated cells vs CQ/B-treated cells

Since C/EBP-β LIP [5] and NO [14, 15] decrease Pgp expression and activity, respectively, we investigated whether the increase in C/EBP-β LIP and NO levels achieved by chloroquine and bortezomib may reduce the efflux of doxorubicin via Pgp. Chloroquine, bortezomib or their combination reduced Pgp mRNA (Fig. 4a) and protein (Fig. 4b) in both MDA-MB-231 and JC cells, in accord with the transcriptional repression of Pgp exerted by LIP [9]. Moreover the lysosome and proteasome inhibitors increased the amount of nitrated - i.e. less active [14, 15] - Pgp on cell surface (Fig. 4b). Consistently, chloroquine and bortezomib, in particular if used in combination, when they maximally increased C/EBP-β LIP and NO levels (Fig. 3b-c), significantly decreased the Pgp ATP-ase activity (Fig. 4c) and the rate of doxorubicin efflux (Fig. 4d).

To verify if the same chemosensitizing effects were maintained in 3D-cultures, a model closer to the in vivo tumor biology and characterized by higher Pgp expression and doxorubicin resistance compared to 2D-cultures [32], we produced 3D-cultures of T47D cells, which were Pgp-negative (Fig. 1a) and doxorubicin-sensitive (Fig. 2b) cells when grown bi-dimensionally. T47D-3D cells increased the expression of Pgp (Additional file 6a), reduced the intracellular doxorubicin retention (Additional file 6b), increased the cell viability in the presence of doxorubicin (Additional file 6c) and lost the ability to induce C/EBP-β LIP in response to the drug (Additional file 6d), behaving like the doxorubicin-resistant/Pgp-positive MDA-MB-231 cells. 3D-cultures had higher lysosome (Additional file 6e) and proteasome (Additional file 6f) activities than 2D-cultures, but they retained the sensitivity to chloroquine and bortezomib. Indeed these two agents reproduced the same effects observed in doxorubicin-resistant/Pgp-positive MDA-MB-231 cells: they increased C/EBP-β LIP/CHOP/TRB3/caspase 3 pathway (Additional file 6 g), decreased Pgp mRNA and protein (Additional file 6 g-h), increased Pgp nitration (Additional file 6 g) in consequence of the increased production of NO (Additional file 6i), restored the intracellular accumulation (Additional file 6j) and cytotoxicity of doxorubicin (Additional file 6 k) to the same levels of doxorubicin-sensitive T47D-2D cells.

### C/EBP-β LIP restores doxorubicin-induced immunogenic cell death in resistant breast cancer cells

**Fig. 5:**
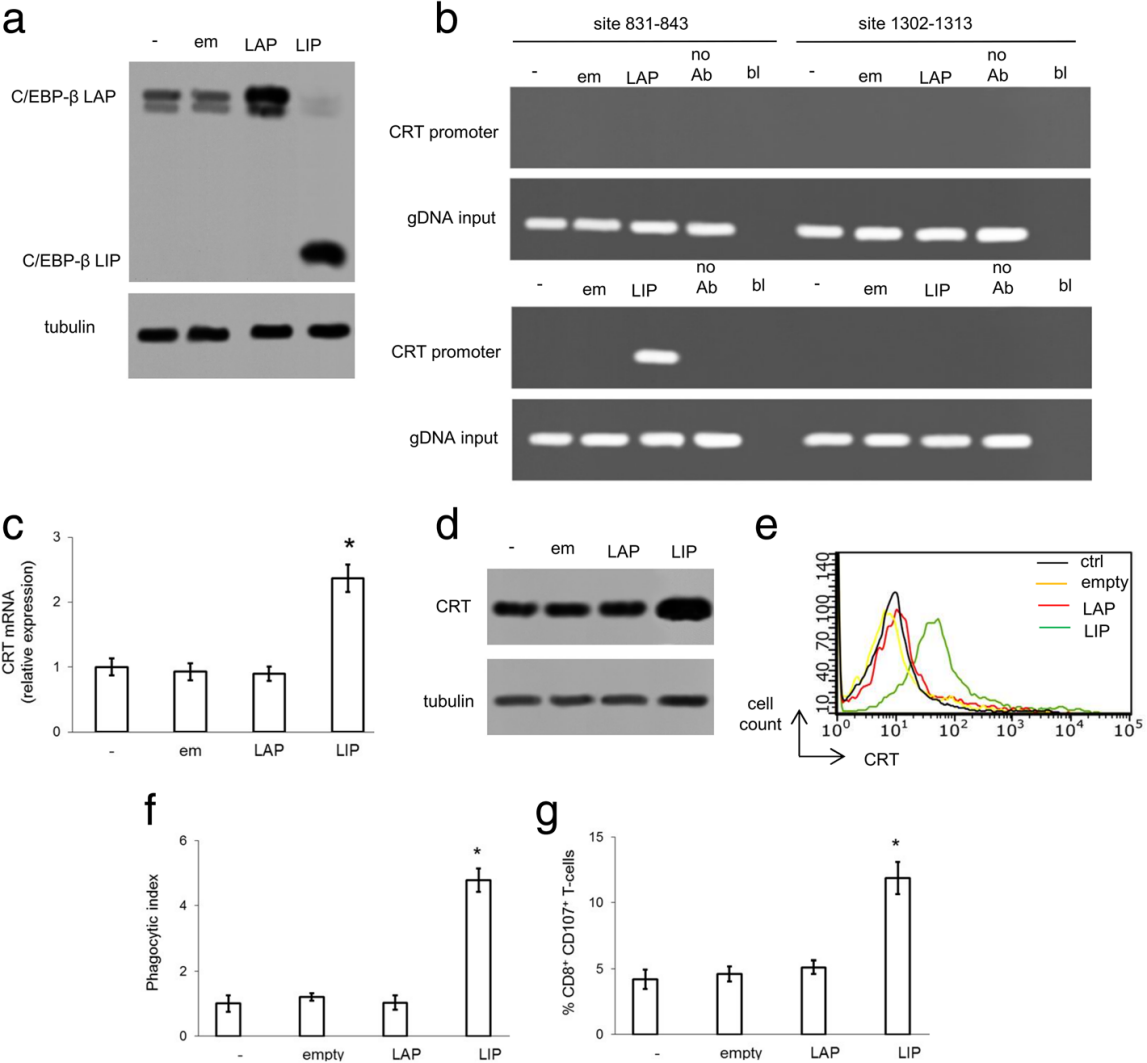
C/EBP-β LIP induces calreticulin expression and immunogenic cell death. MDA-MB-231 cells were left untreated (−, ctrl) or transfected with an empty pcDNA4/TO vector (em/empty), with a pcDNA4/TO expression vector encoding C/EBP-β LAP or C/EBP-β LIP, respectively. **a.** Whole cell lysates were probed with an antibody recognizing both C/EBP-β LAP and LIP isoforms. The expression of β-tubulin was used as control of equal protein loading. The figure is representative of 1 out of 3 experiments. **b.** ChIP was performed to evaluate the binding of LAP or LIP to the *CRT* promoter (sites: 831–843; 1302–1313). no Ab: no anti-C/EBP-β antibody; bl: blank; DNA input: genomic DNA. The figure is representative of 1 out of 3 experiments. **c.** The relative expression of *CRT* gene was measured in triplicates by qRT-PCR. Data are presented as means±SD (n = 3). *p < 0.001: LIP-expressing cells vs all the other experimental conditions. **d.** Whole cell lysates were probed with an anti-CRT antibody. The expression of β-tubulin was used as control of equal protein loading. The figure is representative of 1 out of 3 experiments. **e.** Surface CRT was detected by flow cytometry. The histograms represent the results obtained from 1 out of 3 experiments. **f.** Tumor cells were stained with PKH2-FITC, DC were stained with an anti-HLA-DR-PE antibody. Tumor cells were co-incubated with DC for 24 h. Double-stained cells were counted by flow cytometry. Data are presented as means±SD (*n* = 3). *p < 0.001: LIP-expressing cells vs all the other experimental conditions. **g.** T-lymphocytes were co-cultured with DC after phagocytosis, then incubated with MDA-MB-231 cells. The percentage of CD8^+^CD107^+^T-cells was measured by flow cytometry. Data are presented as means±SD (*n* = 3). *p < 0.001: LIP-expressing cells vs all the other experimental conditions

Since in malignant pleural mesothelioma C/EBP-β LIP transcriptionally activates CRT [27], one of the main ICD effector, we next investigated whether the absent induction of C/EBP-β LIP upon doxorubicin treatment also reduced the pro-immunogenic effects of the anthracycline in resistant TNBC cells. To this aim, we selectively over-expressed C/EBP-β LAP or C/EBP-β LIP in MDA-MB-231 cells (Fig. 5a). C/EBP-β LIP, but not LAP, was bound to the site 831–843 of *CRT* promoter (Fig. 5b). C/EBP-β LIP-overexpressing cells had increased *CRT* mRNA (Fig. 5c), total (Fig. 5d) and surface CRT (Fig. 5e) protein compared with cells transfected with empty vector or overexpressing C/EBP-β LAP. The increase in CRT was paralleled by the increased DC-mediated tumor cell phagocytosis (Fig. 5f) and by the increased expansion of CD8^+^CD107^+^T-lymphocytes incubated with DC that have phagocytized tumor cells (Fig. 5g). The same phenotype was obtained in JC cells transfected with a TetON C/EBP-β LIP-expression vector (Additional file 7a-g).

**Fig. 6:**
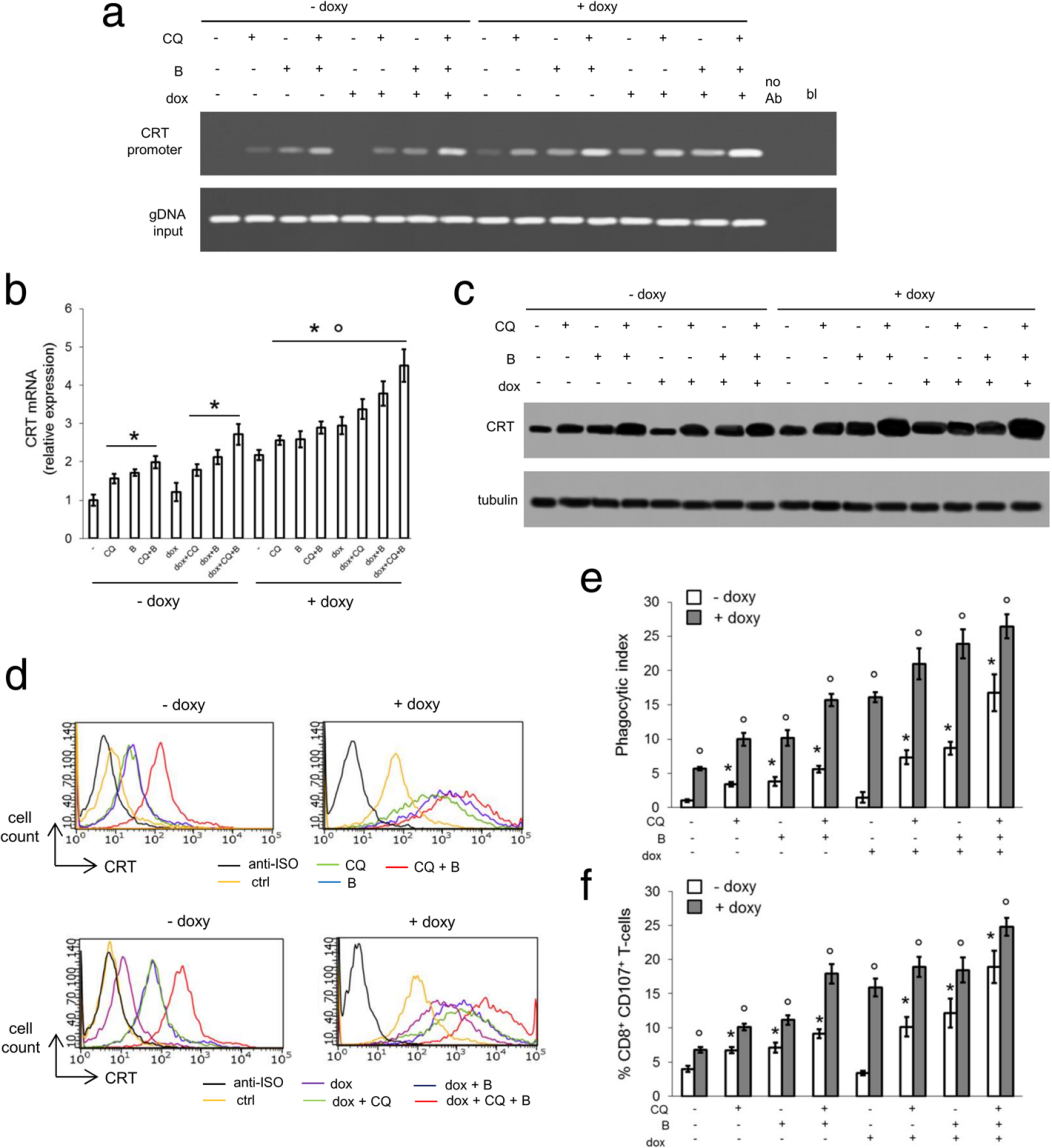
Lysosome and proteasome inhibitors and C/EBP-β LIP overexpression cooperate to induce doxorubicin-triggered immunogenic cell death. MDA-MB-231 cells (−, ctrl) were stably transfected with a doxycycline-inducible vector encoding C/EBP-β LIP. Cells were cultured in the absence (−) or presence (+) of doxycycline (doxy; 1 μg/ml) for 24 h, to induce C/EBP-β LIP. When indicated, cells were co-incubated with the lysosome inhibitor chloroquine (CQ; 1 μM) or with the proteasome inhibitor bortezomib (B; 1 μM), alone or in combination, followed by 5 μM doxorubicin (dox) for further 24 h. **a.** ChIP was performed to evaluate the binding of LIP to the *CRT* promoter (sites: 831–843; 1302–1313). no Ab: no anti-C/EBP-β antibody; bl: blank; DNA input: genomic DNA. The figure is representative of 1 out of 3 experiments. **b.** The relative expression of *CRT* gene was measured in triplicates by qRT-PCR. Data are presented as means±SD (n = 3). *p < 0.01: treatments vs un-induced, untreated (“- doxy,-”) cells; °*p* < 0.05: “+ doxy” cells vs corresponding “- doxy” cells. **c.** Whole cell lysates were probed with an anti-CRT antibody. The expression of β-tubulin was used as control of equal protein loading. The figure is representative of 1 out of 3 experiments. **d.** Surface CRT was detected by flow cytometry. The histograms represent the results obtained from 1 out of 3 experiments. Anti-ISO: anti-isotype antibody. **f.** Tumor cells were stained with PKH2-FITC, DC were stained with an anti-HLA-DR-PE antibody. Tumor cells were co-incubated with DC for 24 h. Double-stained cells were counted by flow cytometry. Data are presented as means±SD (n = 3). *p < 0.002: treatments vs un-induced, untreated (“- doxy,-”) cells; °*p* < 0.001: “+ doxy” cells vs corresponding “- doxy” cells. **g.** T-lymphocytes were co-cultured with DC after phagocytosis, then incubated with MDA-MB-231 cells. The percentage of CD8^+^CD107^+^T-cells was measured by flow cytometry. Data are presented as means±SD (n = 3). *p < 0.001: treatments vs un-induced, untreated (“- doxy,-”) cells; °*p* < 0.001: “+ doxy” cells vs corresponding “- doxy” cells

The combined pharmacological inhibition of lysosome and proteasome in TetON MDA-MB-231 cells - also in the absence of doxycycline - induced C/EBP-β LIP protein (Additional file 8a-b), increased LIP binding to *CRT* promoter (Fig. 6a), *CRT* mRNA (Fig. 6b) and protein (Fig. 6c-d) levels, tumor cell phagocytosis (Fig. 6e) and CD8^+^CD107^+^T-lymphocytes expansion (Fig. 6f). Consistently with the resistance of MDA-MB-231 cells, doxorubicin did not elicit these effects. The combination treatment of chloroquine and bortezomib followed by doxorubicin was more effective than the use of lysosome and proteasome inhibitors alone in inducing C/EBP-β LIP (Additional file 8a-b), triggering the LIP-induced CRT activation and ICD (Fig. 6a-f).

When doxycycline was added to the culture medium to induce C/EBP-β LIP (Additional file 8a-b), LIP transcriptional activity on *CRT* promoter (Fig. 6a-c), CRT translocation (Fig. 6d) and CRT-mediated ICD (Fig. 6e-f) were higher compared to un-induced cells. The maximal efficiency in increasing C/EBP-β LIP (Additional file 8a-b) and CRT-dependent ICD (Fig. 6a-f) was achieved in cells treated with doxycycline (that induces C/EBP-β LIP), chloroquine and bortezomib (that prevent C/EBP-β LIP degradation), and doxorubicin (that elicits ER stress up-regulating endogenous C/EBP-β LIP). These results suggest that maintaining a high level of C/EBP-β LIP, which down-regulate Pgp and up-regulate CRT, fully restores doxorubicin-dependent cell death in vitro, by triggering ER stress-mediated apoptosis and ICD.

### The C/EBP-β LIP effector CHOP mediates ER stress dependent apoptosis and immunogenic cell death in response to chloroquine and bortezomib

**Fig. 7:**
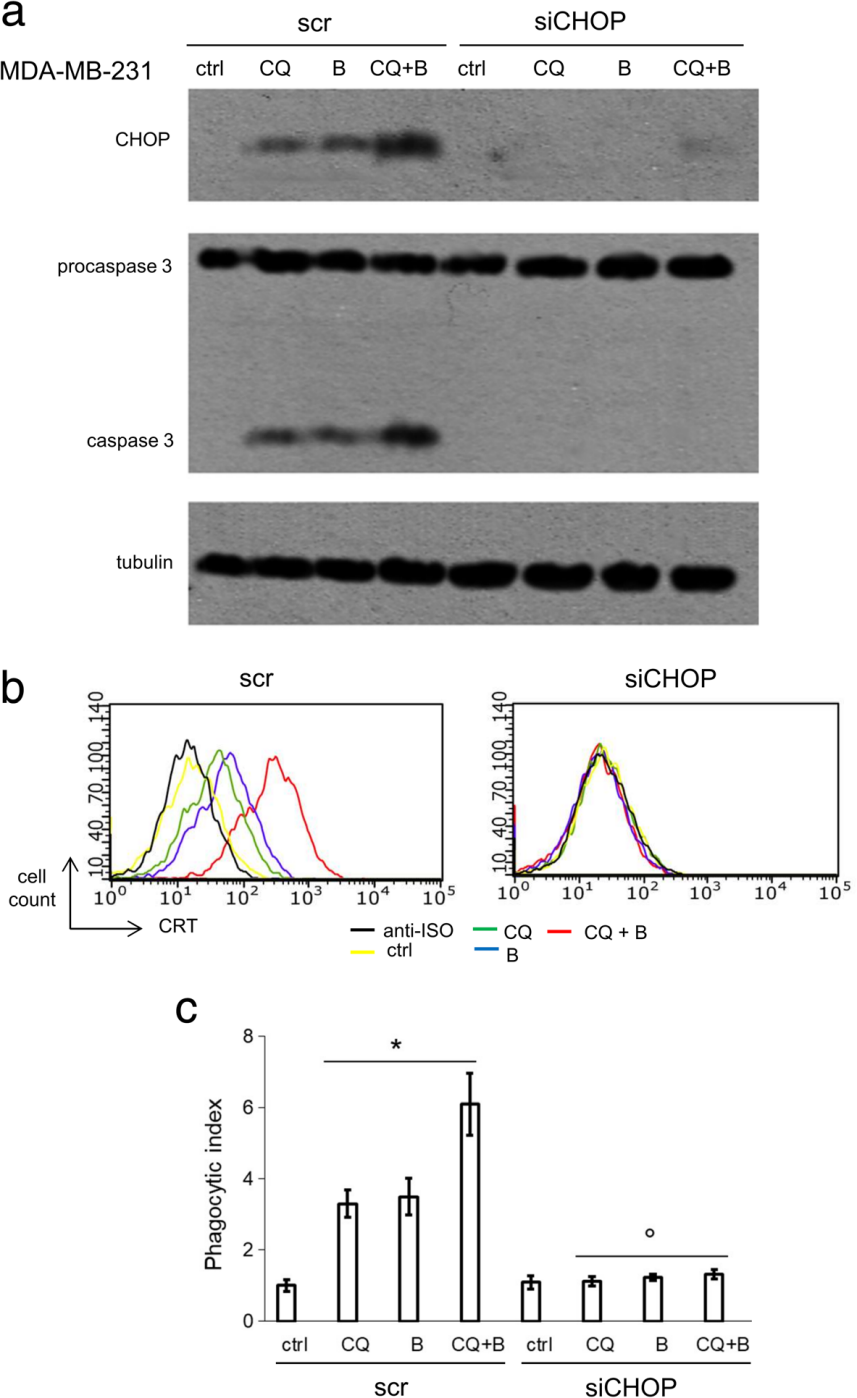
CHOP silencing prevents apoptosis and immunogenic cell death induced by lysosome and proteasome inhibitors. MDA-MB-231 cells were transfected with a non-targeting siRNA (scrambled; scr) or with a CHOP-targeting siRNAs pool (siCHOP). Cells were grown in fresh medium (ctrl) or in a medium containing the lysosome inhibitor chloroquine (CQ; 1 μM) or the proteasome inhibitor bortezomib (B; 1 μM), alone or in combination for 24 h. **a.** Whole cell lysates were probed with the indicated antibodies. The expression of β-tubulin was used as control of equal protein loading. The figure is representative of 1 out of 3 experiments. **b.** Surface CRT was detected by flow cytometry. The histograms represent the results obtained from 1 out of 3 experiments. Anti-ISO: anti-isotype antibody. **c.** Tumor cells were stained with PKH2-FITC, DC were stained with an anti-HLA-DR-PE antibody. Tumor cells were co-incubated with DC for 24 h. Double-stained cells were counted by flow cytometry. Data are presented as means±SD (n = 3). *p < 0.001: CQ/B/CQ + B-treated cells vs “scr, ctrl” cells; °*p* < 0.001: “siCHOP” cells vs corresponding “scr” cells

As detailed above, the activation of C/EBP-β LIP by chloroquine and bortezomib has pleiotropic effects, including increased apoptosis (Fig. 2) and NO production (Fig. 3), decreased Pgp expression and activity (Fig. 4), increased CRT translocation and ICD (Fig. 5, 6). We thus investigated if all these events were dependent on the activation of ER stress-related pathways. To this aim, we transiently silenced CHOP, a key mediator of ER stress-triggered cell death [33–35] and a direct downstream effector of C/EBP-β LIP [36], in MDA-MB-231 cells treated with chloroquine and bortezomib. In cells treated with a non-targeting (scrambled) siRNA, the lysosome and proteasome inhibitor activated caspase 3, while in CHOP-silenced cells they did not (Fig. 7a). We did not detect any differences in the expression and activity of NOS enzymes (Additional file 9a-b), in the expression, nitration and activity of Pgp (Additional file 9c-e), in the amount of *CRT* mRNA and protein (Additional file 9f-g) between scrambled-treated and CHOP-silenced cells. By contrast, the amount of surface CRT and the cell phagocytosis - that were increased in scrambled-treated cells exposed to chloroquine and bortezomib - remained comparable to untreated cells in CHOP-silenced cells (Fig. 7b-c). This experimental set allowed to discriminate the ER stress-dependent and the ER stress-independent events, involved in the rescue of doxorubicin efficacy.

### High C/EBP-β LIP levels restore doxorubicin efficacy in drug-resistant breast cancer xenografts

**Fig. 8:**
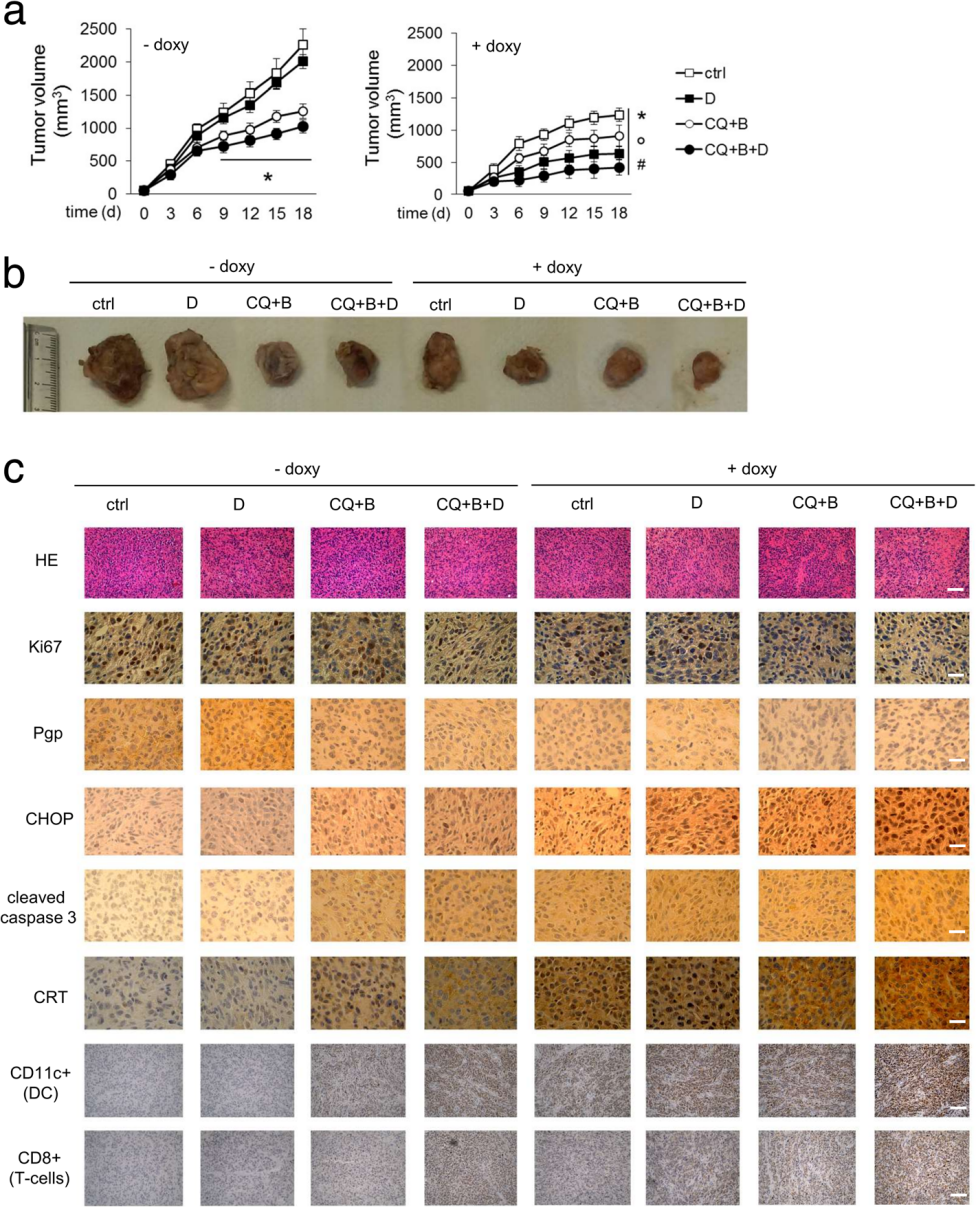
Combination of pharmacological and genetic approaches preserving C/EBP-β LIP restores doxorubicin efficacy in resistant xenografts. JC cells, stably transfected with an inducible expression vector for C/EBP-β LIP (JC TetON LIP) were orthotopically implanted into 6 week-old female balb/C mice. When indicated, animals received 1 mg/ml doxycycline in the drinking water (+ doxy) to induce the intratumor LIP expression. Mice were randomized into 4 groups (*n* = 10 animals/group) and treated once a week for 3 consecutive weeks (days 1, 6, 12 after randomization) as follows: 1) control (ctrl) group, treated with 0.1 ml saline solution intraperitoneally (i.p.); 2) doxorubicin (D) group, treated with 5 mg/kg doxorubicin i.p.; 3) chloroquine+bortezomib (CQ + B) group, treated with 10 mg/kg CQ per os and 0.25 mg/kg bortezomib i.p.; 4) chloroquine+bortezomib+doxorubicin (CQ + B + D) group, treated with chloroquine+bortezomib, followed by doxorubicin after 24 h. **a.** Tumor growth was monitored daily by caliper measurement. Data are presented as means±SD. *p < 0.001: all treatments vs “-doxy, ctrl” group; °*p* < 0.05: “+ doxy” treatments vs corresponding “- doxy” treatments; ^#^*p* < 0.001: “+ dox” treatments vs corresponding “- dox” treatments (days 9–18). **b.** Photographs of representative tumors of each group. **c.** Sections of tumors from each group of animals were stained with hematoxylin and eosin (HE) or with the indicated antibodies. Nuclei were counter-stained with hematoxylin (10× ocular lens, 20× or 63× objective). Bar = 10 μm

In line with the in vitro results, the growth of orthotopically implanted TetON JC tumors, that were completely insensitive to doxorubicin, was reduced by the combination of chloroquine and bortezomib (Fig. 8a-b). In the absence of C/EBP-β LIP induction, doxorubicin slightly increased the anti-tumor effects of chloroquine and bortezomib. When C/EBP-β LIP was induced, chloroquine and bortezomib produced a stronger reduction in tumor growth, and the subsequent addition of doxorubicin elicited a further decrease (Fig. 8a-b).

Un-induced tumors treated with chloroquine and bortezomib and C/EBP-β LIP-induced tumors not treated with chloroquine and bortezomib had a comparable decrease in tumor cell proliferation, and a comparable increase in ER stress and apoptosis, as suggested by the staining for Ki67, CHOP and active caspase 3. In parallel, tumors showed decreased Pgp, increased CRT positivity, increased intratumor infiltration of DC and cytotoxic T-lymphocytes (Fig. 8c; Additional file 10a). Also the production of IFN-γ from draining lymph nodes, a marker of local immune system activation, was increased (Additional file 10b). Tumors with induced C/EBP-β LIP, treated with chloroquine and bortezomib followed by doxorubicin, displayed a further reduction in proliferation and Pgp expression, as well as a further increase in ER stress, apoptosis, CRT positivity, intratumor DC, cytotoxic T-lymphocytes (Fig. 8c, Additional file 10a), IFN-γ production (Additional file 10b).

Of note, the combined treatments did not induce signs of systemic toxicity, nor worsen the cardiac damage induced by doxorubicin, according to the animals’ hematochemical parameters (Additional file 11).

### C/EBP-β LIP and calreticulin are both necessary to restore immunogenic cell death in doxorubicin-resistant breast cancers

**Fig. 9:**
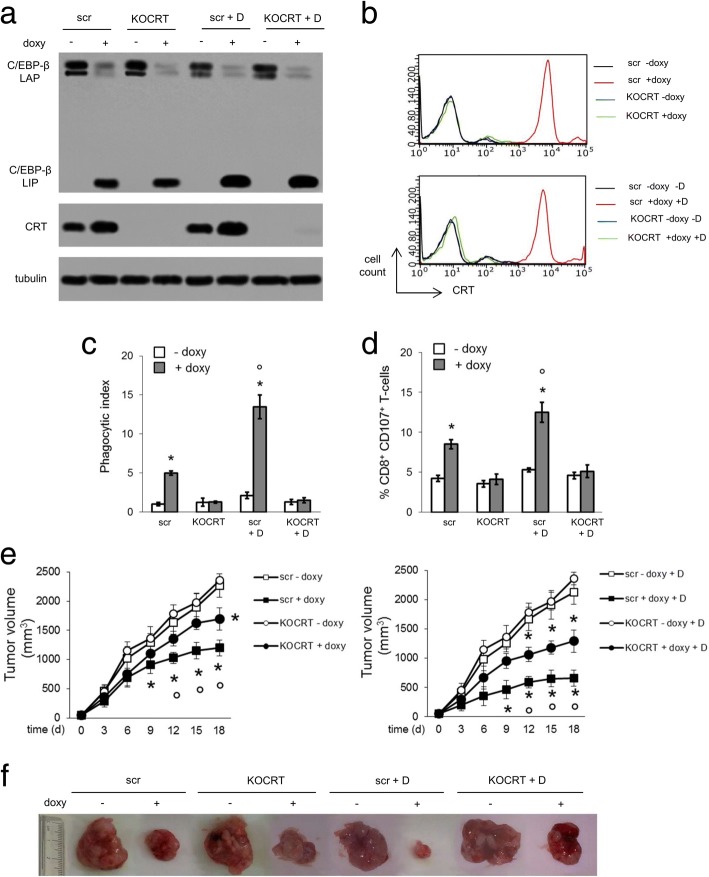
Doxorubicin-triggered immunogenic cell death is dependent either on C/EBP-β LIP or calreticulin. JC TetON LIP cells, transduced with a non-targeting (scrambled, scr) vector or with a knocking-out calreticulin (KOCRT) vector, were cultured in the absence (− doxy) or presence (+ doxy) of doxycycline (1 μg/ml) for 24 h, to induce C/EBP-β LIP. When indicated, 5 μM doxorubicin (D) was added for 24 h further. **a.** Whole cell lysates were probed with the indicated antibodies. The expression of β-tubulin was used as control of equal protein loading. The figure is representative of 1 out of 3 experiments. **b.** Surface CRT was detected by flow cytometry. The histograms represent the results obtained from 1 out of 3 experiments. **c.** Tumor cells were stained with PKH2-FITC, DC were stained with an anti-HLA-DR-PE antibody. Tumor cells were co-incubated with DC for 24 h. Double-stained cells were counted by flow cytometry. Data are presented as means±SD (n = 3). *p < 0.001: “+ doxy” cells vs “- doxy” cells; °p < 0.001: “KOCRT” cells vs “scr” cells. **d.** T-lymphocytes were co-cultured with DC after phagocytosis, then incubated with JC cells. The percentage of CD8^+^CD107^+^T-cells was measured by flow cytometry. Data are presented as means±SD (n = 3). *p < 0.001: “+ doxy” cells vs “- doxy” cells; °*p* < 0.001: “KOCRT” cells vs “scr” cells. **e.** Cells were implanted orthotopically into 6 week-old female balb/C mice. When indicated, animals received 1 mg/ml doxycycline in the drinking water (+ doxy) to induce the intratumor LIP expression. Mice were randomized into 8 groups (*n* = 8 animals/group) treated on days 1, 6, 12 after randomization: 4 groups received 0.1 ml saline solution i.p. (left panel), 4 groups received 5 mg/kg doxorubicin (D) i.p. (right panel). Tumor growth was monitored daily by caliper measurement. Data are presented as means±SD. *p < 0.001: “+ doxy” cells vs “- doxy” cells; °*p* < 0.001: “KOCRT” cells vs “scr” cells. **f.** Photographs of representative tumors of each group

The immunogenic effect of doxorubicin in vitro and in vivo was strictly dependent on CRT: indeed, in JC clones with induced C/EBP-β LIP but knocked-out for CRT (Fig. 9a), doxorubicin was unable to induce CRT translocation on cell surface (Fig. 9b), tumor cell phagocytosis (Fig. 9c) and CD8^+^CD107^+^T-lymphocytes expansion (Fig. 9d). Both KO-CRT and wild-type tumors had a reduced growth in the presence of C/EBP-β LIP induction (Fig. 9e-f). However, while in C/EBP-β LIP-induced/CRT-wild-type tumors, doxorubicin strongly reduced tumor growth, the drug had significantly lower anti-tumor efficacy in C/EBP-β LIP-induced/KO-CRT tumors (Fig. 9d-e). These results suggested that both C/EBP-β LIP and CRT are necessary to restore the ICD-induced by doxorubicin against drug-resistant/Pgp-positive breast cancers.

## Discussion

Anthracycline-based chemotherapy is one of the first-line treatment in TNBC, but half of the patients develop resistance [2]. Effective strategies of chemosensitization are still an unmet need.

The resistance to doxorubicin is mostly mediated by Pgp, which limits the intracellular accumulation of the drug and the possibility of exerting its pleiotropic cytotoxic mechanisms, such as increasing NO levels, inducing ER stress and ICD [5, 16, 17, 20]. Since Pgp can be inhibited at transcriptional level by C/EBP-β LIP [5] and at post-translational level by NO [14, 15], we set up a strategy that increases at the same time C/EBP-β LIP and NO upon treatment with doxorubicin, in order to downregulate expression and activity of Pgp and overcome the resistance to the drug in TNBC.

By screening different breast cancer cell lines, we found that TNBC human and murine doxorubicin-resistant/Pgp-positive cells did not induce C/EBP-β LIP in response to doxorubicin, differently from doxorubicin-sensitive/Pgp-negative cells. The absence of LIP, as documented in other chemoresistant tumors [5, 27], was due to its mono- and poly-ubiquitination, followed by lysosomal and proteasomal degradation. Indeed, doxorubicin-resistant/Pgp-positive TNBC MDA-MB-231 and JC cells displayed the highest activity of lysosome and proteasome, coupled with the lowest induction of C/EBP-β LIP upon doxorubicin treatment. We thus employed lysosome and proteasome inhibitors as pharmacological tools able to prevent C/EBP-β LIP degradation, either in basal conditions or upon doxorubicin treatment.

The elevated expression of the lysosomal enzyme cathepsin D has been already correlated with breast cancer progression [37], opening the way to the design of cathepsin D inhibitors as potential anti-tumor agents [38]. In the present work, we used chloroquine, a FDA-approved lysosome and autophagosome inhibitor [21] that exerts anti-tumor effects against TNBC stem cells [39] and enhances the ER stress-dependent cell death by inhibiting lysosome activity [40, 41].

Also the high activity of proteasome has been correlated with poor prognosis in TNBC patients and poor response to chemotherapy in vitro [42]. Proteasome inhibitors are under evaluation as new therapeutic options in TNBC patients [43]. Our work may provide the rationale for the combined use of lysosome and proteasome inhibitors, in association with first-line therapy doxorubicin, against resistant TNBC as a new combination treatment that activates pleiotropic mechanisms of cell killing.

First, by increasing C/EBP-β LIP/CHOP/TRB3/caspase 3 axis, chloroquine and bortezomib induced a ER stress-dependent apoptosis with at least two mechanism. Indeed, on the one hand chloroquine and bortezomib prevented the lysosomal and proteasomal degradation of LIP, as demonstrated by the abrogation of their effects in cells co-incubated with lysosome or proteasome activators. On the other hand, chloroquine, bortezomib and doxorubicin all increase NO production [17, 29, 30]. NO in turn induces ER stress [19], and activates CHOP and caspase 3 [44]. Since the NO donor SNP induced LIP as chloroquine and bortezomib did, while the NO scavenger carboxy-PTIO abrogated the effects of the lysosome and proteasome inhibitors, we propose that chloroquine and bortezomib increased C/EBP-β LIP either by preventing its degradation via lysosome and proteasome, or by eliciting a NO-dependent ER stress. The combination treatment with chloroquine, bortezomib and doxorubicin boosted the activation of ER stress-dependent cell death in TNBC cells, restoring one of the cytotoxic mechanisms of doxorubicin.

Second, by increasing C/EBP-β LIP, chloroquine and bortezomib down-regulated Pgp expression and increased CRT. The down-regulation of Pgp allowed doxorubicin to reach an intracellular concentration sufficient to activate the pro-apoptotic C/EBP-β LIP/CHOP/TRB3/caspase 3 pathway and to increase the synthesis of NO. The activation of ER stress-dependent cell death pathway and the increase in NO promote the translocation of CRT from ER to plasma-membrane. The up-regulation of CRT elicited by C/EBP-β LIP at transcriptional level further enhanced this process, contributing to restore ICD and to re-establish a second cytotoxic mechanisms of doxorubicin.

Third, chloroquine and bortezomib increased the synthesis of NO, a non-competitive inhibitor of Pgp [14, 15], that reduced the Vmax of doxorubicin efflux through the transporter. The role of NO in cancer is still controversial, since it can act as a tumorigenic or anti-cancer agent [45, 46], an activator or suppressor of the DC and T-lymphocytes [47], depending on the concentration and temporal production. The most studied NOS enzyme in TNBC has been NOS II, a negative prognostic factor [48] and an inducer of resistance to docetaxel [49]. As demonstrated by Dávila-González and coworkers, NOS II inhibition, coupled to a constant expression of NOS III, sensitized TNBC cells to docetaxel and activated the ER stress-dependent pro-apoptotic transducers such as CHOP [49]. This situation closely mimics the scenario of TNBC cells treated with chloroquine and bortezomib, where NOS II was undetectable, NOS III expression was constant, cells were re-sensitized to doxorubicin and activated CHOP-dependent pathways. Of note, docetaxel is a substrate of Pgp as doxorubicin [7]: it is possible that the chemosensitization observed by Dávila-González and coworkers was in part due to the reduced efflux of docetaxel, consequent to the inhibition of Pgp elicited by NO. This hypothesis is supported by our findings in TNBC cells treated with chloroquine and bortezomib, that had increased NO synthesis, reduced Pgp activity, decrease efflux of the Pgp substrate doxorubicin.

Given the pleiotropic effects exerted by the combination of chloroquine and bortezomib, we next investigated if all the observed events were due to the C/EBP-β LIP-mediated induction of ER stress. To this aim, we transiently silenced CHOP, a downstream effector of C/EBP-β LIP [36] and a promoter of ER stress-dependent cell death [33–35]. The results obtained in silenced cells indicated that the activation of the caspase 3 elicited by chloroquine and bortezomib, the translocation of CRT and the consequent ICD, were dependent on ER stress. These data were in accord with several evidences demonstrating that ER stress induces apoptosis via CHOP [50], triggers the surface translocation of CRT and the CRT-mediated phagocytosis [3, 6]. By contrast, ER stress was not responsible for the increased production of NO, the reduction in Pgp expression and activity, the up-regulation of CRT gene. Indeed, the increase in NO was due to the higher expression of NOS I induced by bortezomib and to the higher activity of NOS III induced by chloroquine. The down-regulation of Pgp and the up-regulation of CRT were caused by the activity of C/EBP-β LIP as transcription factor. The reduced activity of Pgp was consequent to the increased production of NO that nitrated tyrosine residues critical for the pump’s catalytic activity.

We can conclude that the effects of chloroquine and bortezomib were either ER stress-dependent or independent, but they all triggered virtuous circuitries that effectively overcome the resistance to doxorubicin in Pgp-positive TNBC cells.

The effects of choloroquine and bortezomib observed in vitro were well reproduced in the preclinical model of doxorubicin-resistant/Pgp-positive JC tumors.

The use of chloroquine and bortezomib in TNBC is not new. It has been reported that chloroquine chemosensitizes TNBC-derived xenografts at 50 mg/kg [49], i.e. five-fold the concentration used in the present study. Bortezomib partially reduced tumor growth in TNBC patient-derived xenografts at 0.75 mg/kg [51], i.e. three-fold the dosage used in our in vivo protocol. In chemoresistant TNBC patients bortezomib achieved a partial response, followed by disease recurrence [42]. The novelty of our approach is the use of lower doses of chloroquine and bortezomib in combination with doxorubicin. Such combination therapy did not elicit systemic toxicity, but it reduced tumor growth, increased intratumor ER stress, apoptosis, CRT expression, DC and CD8^+^T-lymphocyte infiltration. The intratumor activation of C/EBP-β LIP further enhanced the responses elicited by the chloroquine /bortezomib/doxorubicin combination, confirming that C/EBP-β LIP and its downstream effectors (such as CHOP and caspase 3) are critical factors in overcoming doxorubicin resistance.

An anti-tumor abundant immune-infiltrate, indicated by high CD8^+^T-lymphocytes and low T-regulatory cells, is a strong predictor of good response to chemotherapy in breast cancer patients [52, 53], suggesting that part of the anti-tumor effects of anthracyclines is mediated by the engagement of the host immune system. In agreement with this hypothesis, knocking-out CRT in tumors with induced C/EBP-β LIP completely abrogated the doxorubicin-induced ICD and reduced doxorubicin anti-tumor efficacy, indicating that both C/EBP-β LIP and CRT are necessary for a full rescue of doxorubicin efficacy in resistant tumors.

## Conclusions

**Fig. 10:**
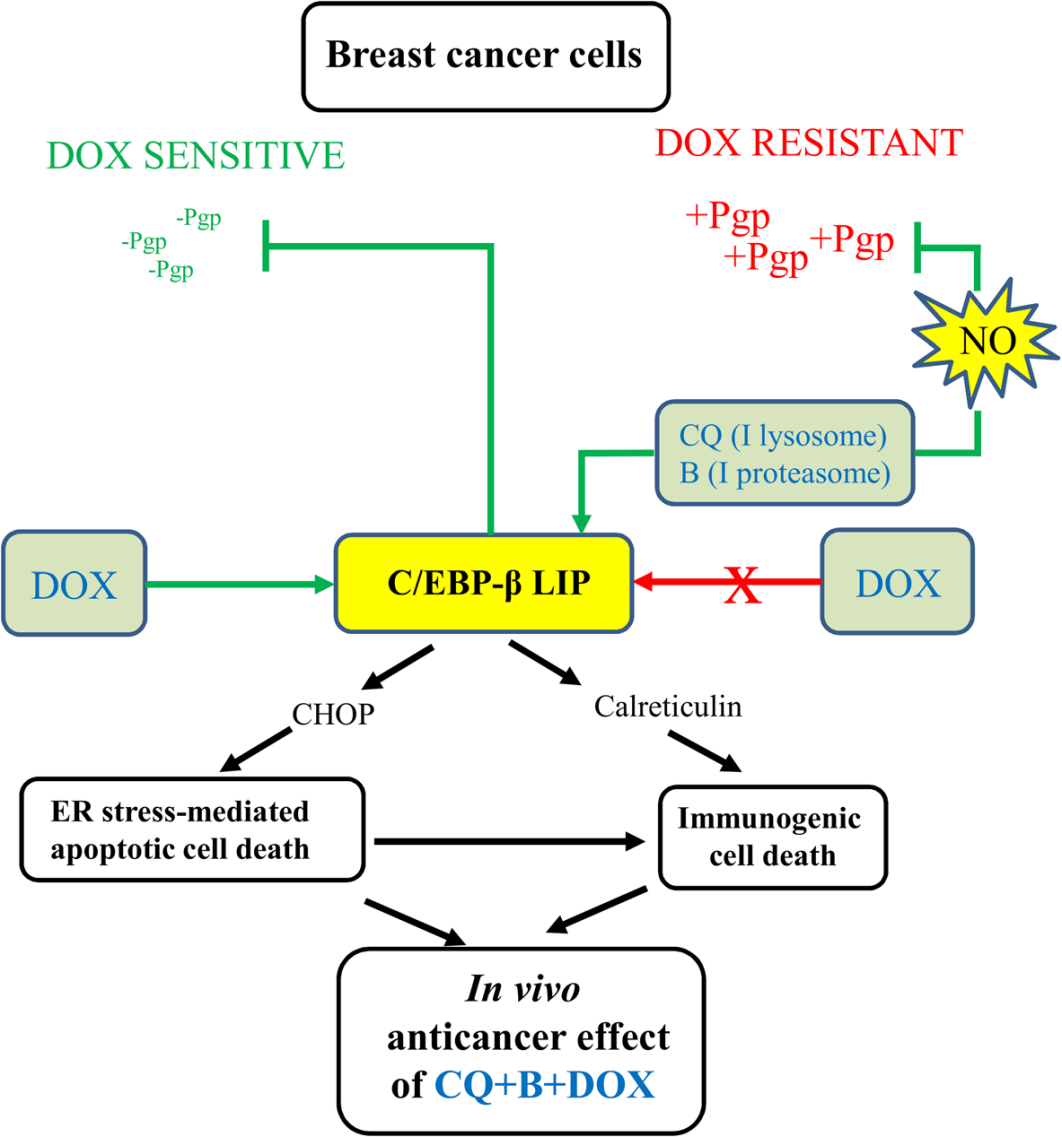
Proposed molecular circuitries linking C/EBP-β LIP, NO and Pgp in breast cancer. Breast cancer doxorubicin-sensitive cells have high C/EBP-β LIP/CHOP/TRB3/caspase 3-axis, low expression of P-glycoprotein (Pgp) and high expression of calreticulin, consequent to the high C/EBP-β LIP levels. In these cells, doxorubicin (DOX) reaches intracellular concentrations sufficient to induce ER stress-dependent cell death and immunogenic cell death. By contrast, doxorubicin-resistant cells have high lysosome and proteasome activities that down-regulates C/EBP-β LIP, prevent the activation of C/EBP-β LIP/CHOP/TRB3/caspase 3-axis, decreases calreticulin expression and nitric oxide (NO) production. These events determine high expression and activity of Pgp and abrogate the efficacy of doxorubicin. The combined inhibition (I) of lysosome and proteasome with chloroquine (CQ) and bortezomib (B) restores the C/EBP-β LIP/CHOP/TRB3/caspase 3-axis and the synthesis of NO, lowering expression and activity of Pgp, increasing transcription and translocation to the plasma-membrane of calreticulin. This rewiring restores the mechanisms of doxorubicin’s induced cell death via ER stress and immunogenic cell death, overcoming drug resistance in vitro and in vivo

In summary, we suggest that the high activities of lysosome and proteasome – that down-regulated C/EBP-β LIP and LIP/CHOP/TRB3/caspase 3-axis – and the low production of NO are hallmarks of Pgp-positive TNBC and inducers of doxorubicin resistance. The combined use of chloroquine and bortezomib increases at the same time C/EBP-β LIP and NO levels: this condition restores the ER-dependent/CHOP-mediated apoptosis, down-regulates Pgp expression and activity, increases CRT expression and translocation to the plasma-membrane, re-activates the DC/CD8^+^T-lymphocyte response against the tumor upon doxorubicin treatment (Fig. 10). Since either chloroquine or bortezomib are already approved for clinical use, our work may open the way to their repurposing as adjuvant agents in patients with TNBC resistant to anthracyclines.

## Additional files

Additional file 1: **Table S1** IC_50_ to doxorubicin in the cell lines analyzed. (DOCX 14 kb)

Additional file 2: **Figure S1.** Dose-dependence cell viability upon treatment with chloroquine and bortezomib. (DOCX 2931 kb)

Additional file 3: **Figure S2.** Chloroquine and bortezomib inhibition of lysosome and proteasome activity. (DOCX 1074 kb)

Additional file 4: **Figure S3.** Chloroquine and bortezomib increase nitrite levels. (DOCX 721 kb)

Additional file 5: **Figure S4.** Effect of chloroquine, bortezomib, sodium nitroprusside and carboxy-PTIO on C/EBP-β LIP/CHOP/TRB3/caspase 3 axis in murine JC cells. (DOCX 799 kb)

Additional file 6: **Figure S5.** Chloroquine and bortezomib reversion of doxorubicin resistance in 3D-cultures. (DOCX 3366 kb)

Additional file 7: **Figure S6.** Set up and validation of an inducible C/EBP-β LIP expression system in Pgp-positive/doxorubicin-resistant JC cells. (DOCX 1224 kb)

Additional file 8: **Figure S7.** C/EBP-β LIP levels in TetON MDA-MB-231 cells, treated with chloroquine, bortezomib and doxorubicin. (DOCX 1723 kb)

Additional file 9: **Figure S8.** Effects of CHOP silencing on nitric oxide production, Pgp expression and activity, calreticulin expression. (DOCX 3230 kb)

Additional file 10: **Figure S9.** Immunohistochemical and immunological parameters of mice exposed to chloroquine, bortezomib and doxorubicin. (DOCX 2475 kb)

Additional file 11: **Table S2** Hematochemical parameters of animals treated with doxorubicin, chloroquine and bortezomib, in the presence of intratumorally induced C/EBP-β LIP. (DOCX 16 kb)

## Abbreviations

ANOVA: One-way analysis of variance
BCRP: Breast cancer resistance protein
BSA: Bovine serum albumin
C/EBP: CAAT/enhancer binding protein
ChIP: Chromatin immunoprecipitation
CHOP: C/EBP homologous protein
CRT: Calreticulin
DAPI: 4′,6-diamidino-2-phenylindole dihydrochloride
DC: Dendritic cell
ER: Endoplasmic reticulum
FBS: Fetal bovine serum
FITC: Fluorescein isothyocyanate
GFP: Green fluorescence protein
ICD: Immunogenic cell death
KO: Knock-out
MRP1: Multidrug resistant protein 1
NO: Nitric oxide
NOS: Nitric oxide synthase
PE: Phycoerythrin
PFA: Paraformaldehyde
Pgp: P-glycoprotein
qRT-PCR: Quantitative Real Time-PCR
RLU: Relative luminescence units
TBS: Tris-buffered saline
TNBC: Triple negative breast cancer
TRB3: Tribbles 3
