# Supplementary material for: Increasing intratumor C/EBP-β LIP and nitric oxide levels overcome resistance to doxorubicin in triple negative breast cancer
Source: J Exp Clin Cancer Res. 2018 Nov 27;37:286. doi: 10.1186/s13046-018-0967-0 (PMC6258159; doi:10.1186/s13046-018-0967-0)
Supplement: Supplementary file 1 — Table S1 IC50 to doxorubicin in the cell lines analyzed. (DOCX 14 kb) [file 13046_2018_967_MOESM1_ESM.docx]

**Additional file 1: Table S1. IC_50_ to doxorubicin in the cell lines analyzed**

| **Cell line** | **IC_50_ (µM)** |
| --- | --- |
| MCF10A | 1.81 + 0.13 |
| MCF7 | 2.86 + 0.27 |
| SKBR3 | 2.21 + 0.31 |
| T47D | 2.09 + 0.31 |
| MDA-MB-231 | 10.03 + 0.62 |
| JC | 25.09 + 3.27 |
| TUBO | 9.93 + 1.12 |

IC_50_ was calculated as detailed in the Materials and methods section. Data are means±SD (n=4)
