# Supplementary material for: Increasing intratumor C/EBP-β LIP and nitric oxide levels overcome resistance to doxorubicin in triple negative breast cancer
Source: J Exp Clin Cancer Res. 2018 Nov 27;37:286. doi: 10.1186/s13046-018-0967-0 (PMC6258159; doi:10.1186/s13046-018-0967-0)
Supplement: Supplementary file 2 — Figure S1. Dose-dependence cell viability upon treatment with chloroquine and bortezomib. (DOCX 2931 kb) [file 13046_2018_967_MOESM2_ESM.docx]

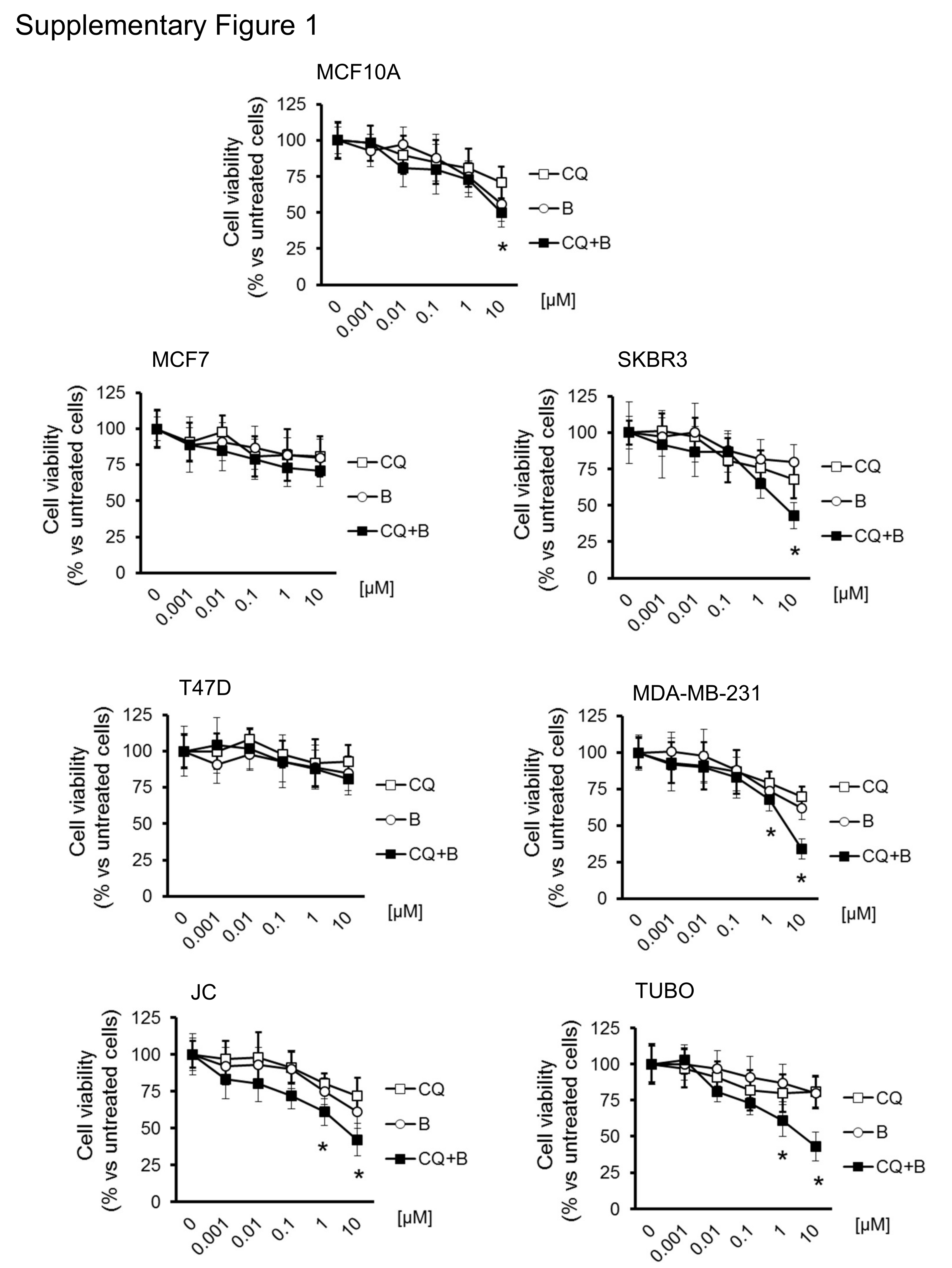


**Additional file 2: Figure S1. Dose-dependent cell viability upon treatment with chloroquine and bortezomib**

Cells were seeded in quadruplicates in 96-well plates, treated for 72 h with chloroquine (CQ) or/and bortezomib (B) at scalar concentrations (from 10^-6^ to 10^-2^M), then viability was measured by a chemiluminescence-based method. Data are presented as means±SD (n=4). *p<0.05: treated cells vs. untreated cells.
