# Supplementary material for: Increasing intratumor C/EBP-β LIP and nitric oxide levels overcome resistance to doxorubicin in triple negative breast cancer
Source: J Exp Clin Cancer Res. 2018 Nov 27;37:286. doi: 10.1186/s13046-018-0967-0 (PMC6258159; doi:10.1186/s13046-018-0967-0)
Supplement: Supplementary file 3 — Figure S2. Chloroquine and bortezomib inhibition of lysosome and proteasome activity. (DOCX 1074 kb) [file 13046_2018_967_MOESM3_ESM.docx]

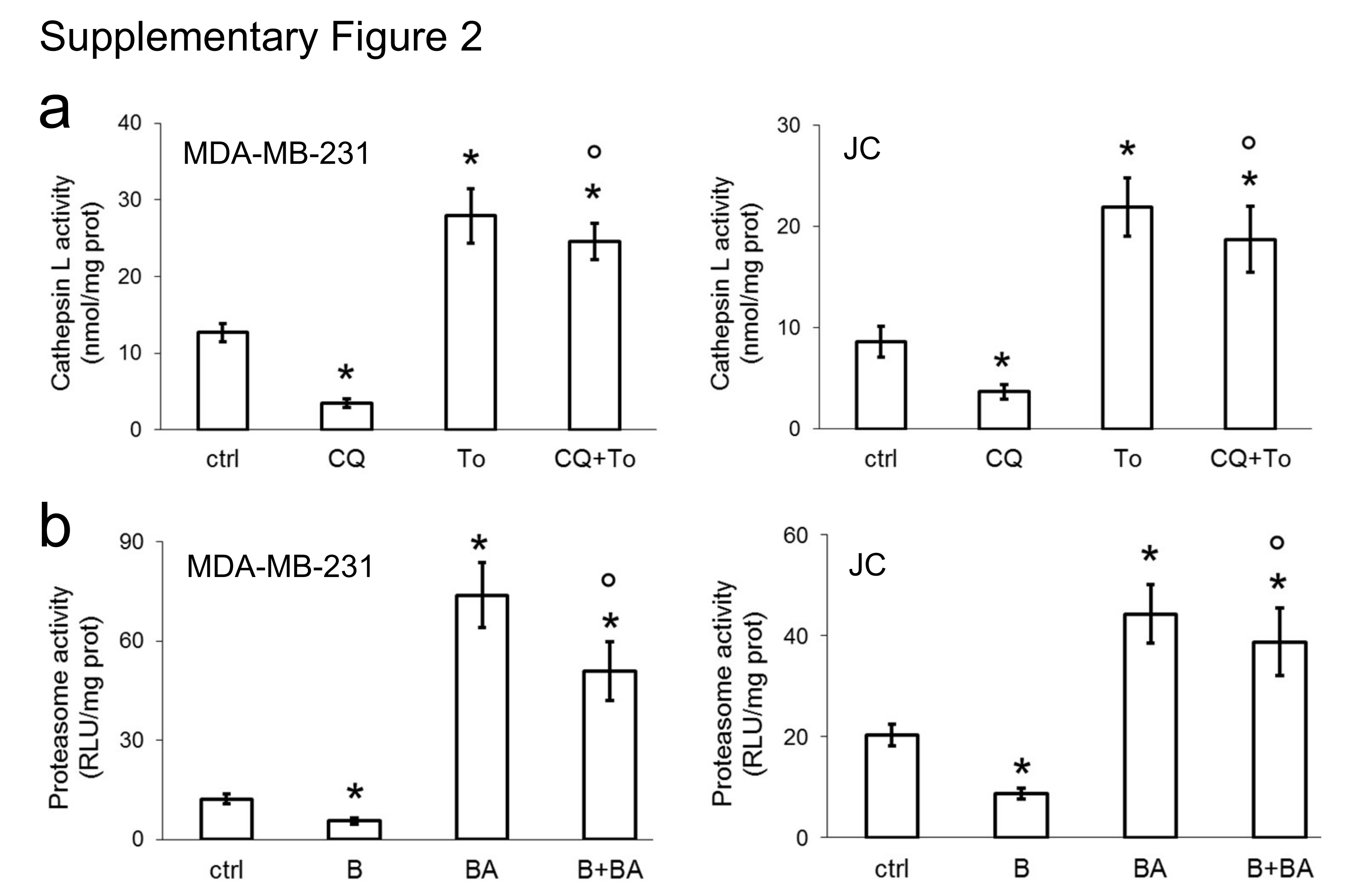


**Additional file 3: Figure S2 Chloroquine and bortezomib inhibit lysosome and proteasome activity**

MDA-MB-231 and JC cells were cultured in the absence (ctrl) or presence of the lysosome inhibitor chloroquine (CQ; 1 μM for 24 h) or the proteasome inhibitor bortezomib (B; 1 μM for 24 h). When indicated, the lysosome activator torin-1 (To; 1 μM) or the proteasome activator betulinic acid (BA; 10 μM) were added, as positive controls. **a**. Lysosome activity was analyzed in duplicates by a spectrophotometric assay. Data are presented as means±SD (n=3). *p<0.001: ctrl vs treated cells; °p<0.001: CQ+To-treated cells vs To-treated cells. **b.** Proteasome activity was analyzed in duplicates by a chemiluminescence-based assay. Data are presented as means±SD (n=3). *p<0.001: ctrl vs treated cells; °p<0.001: B+Ba-treated cells vs B-treated cells.
