# Supplementary material for: Increasing intratumor C/EBP-β LIP and nitric oxide levels overcome resistance to doxorubicin in triple negative breast cancer
Source: J Exp Clin Cancer Res. 2018 Nov 27;37:286. doi: 10.1186/s13046-018-0967-0 (PMC6258159; doi:10.1186/s13046-018-0967-0)
Supplement: Supplementary file 4 — Figure S3. Chloroquine and bortezomib increase nitrite levels. (DOCX 721 kb) [file 13046_2018_967_MOESM4_ESM.docx]

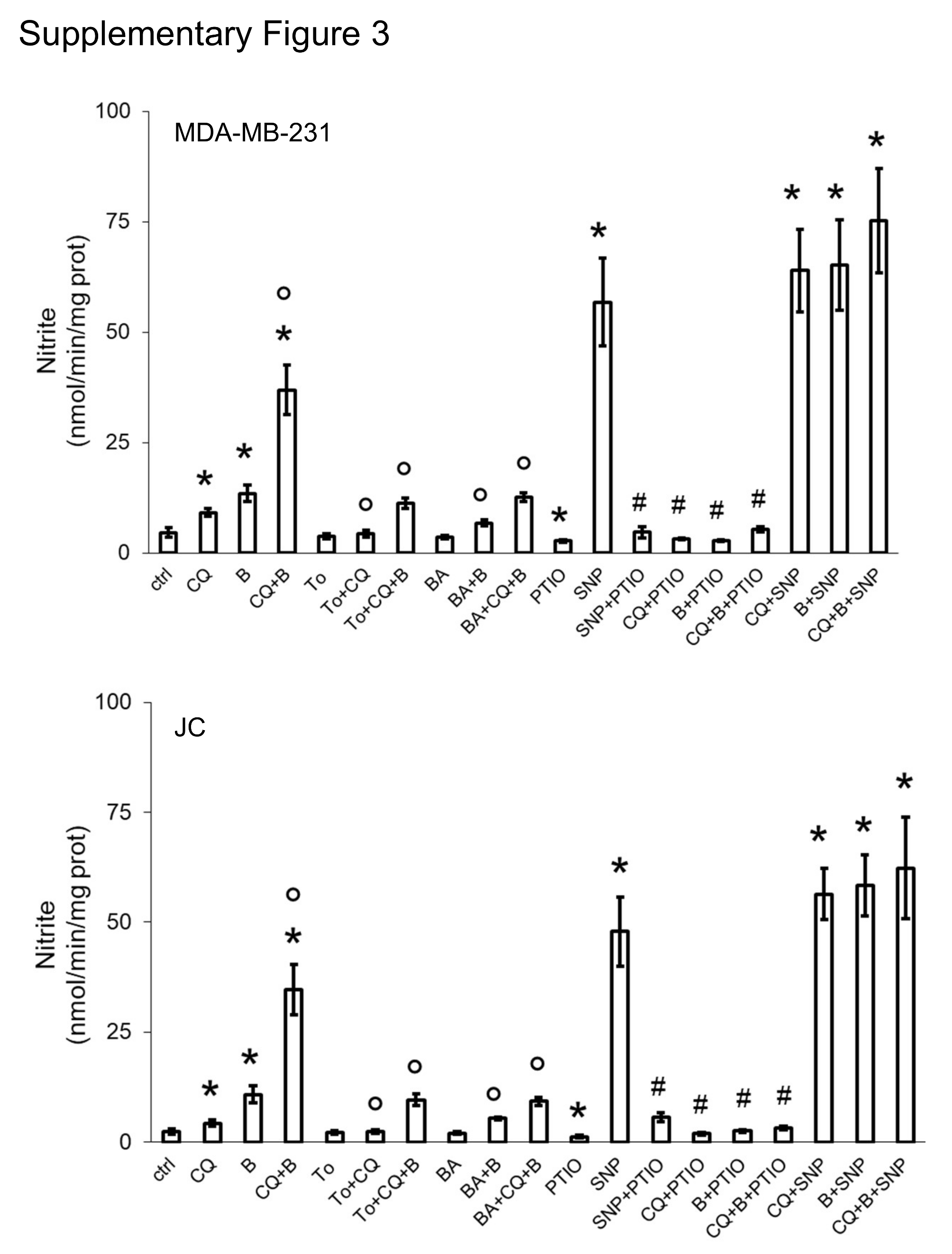


**Additional file 4: Figure S3. Chloroquine and bortezomib increase nitrite levels**

Cells were cultured for 24 h in the absence (ctrl) or presence of the lysosome inhibitor chloroquine (CQ; 1 μM), the proteasome inhibitor bortezomib (B; 1 μM), the lysosome activator torin-1 (To; 1 μM), the proteasome activator betulinic acid (BA; 10 μM), the NO donor sodium nitroprusside (SNP; 10 μM), the NO scavenger carboxy-PTIO (PTIO; 100 μM), alone or co-incubated in different combinations. Nitrite levels in the supernatants were measured in triplicates by a spectrophotometric assay. Data are mean±SD (n=3). *p<0.02: treated cells vs ctrl cells; °p<0.002: To+CQ/To+CQ+B-treated cells vs To-treated cells; BA+B/BA+CQ+B-treated cells vs BA-treated cells; ^#^p<0.001: SNP+PTIO/CQ+PTIO/B+PTIO/CQ+B+PTIO-treated cells vs SNP/CQ/B/CQ+B-treated cells.
