# Supplementary material for: Increasing intratumor C/EBP-β LIP and nitric oxide levels overcome resistance to doxorubicin in triple negative breast cancer
Source: J Exp Clin Cancer Res. 2018 Nov 27;37:286. doi: 10.1186/s13046-018-0967-0 (PMC6258159; doi:10.1186/s13046-018-0967-0)
Supplement: Supplementary file 5 — Figure S4. Effect of chloroquine, bortezomib, sodium nitroprusside and carboxy-PTIO on C/EBP-β LIP/CHOP/TRB3/caspase 3 axis in murine JC cells. (DOCX 799 kb) [file 13046_2018_967_MOESM5_ESM.docx]

**
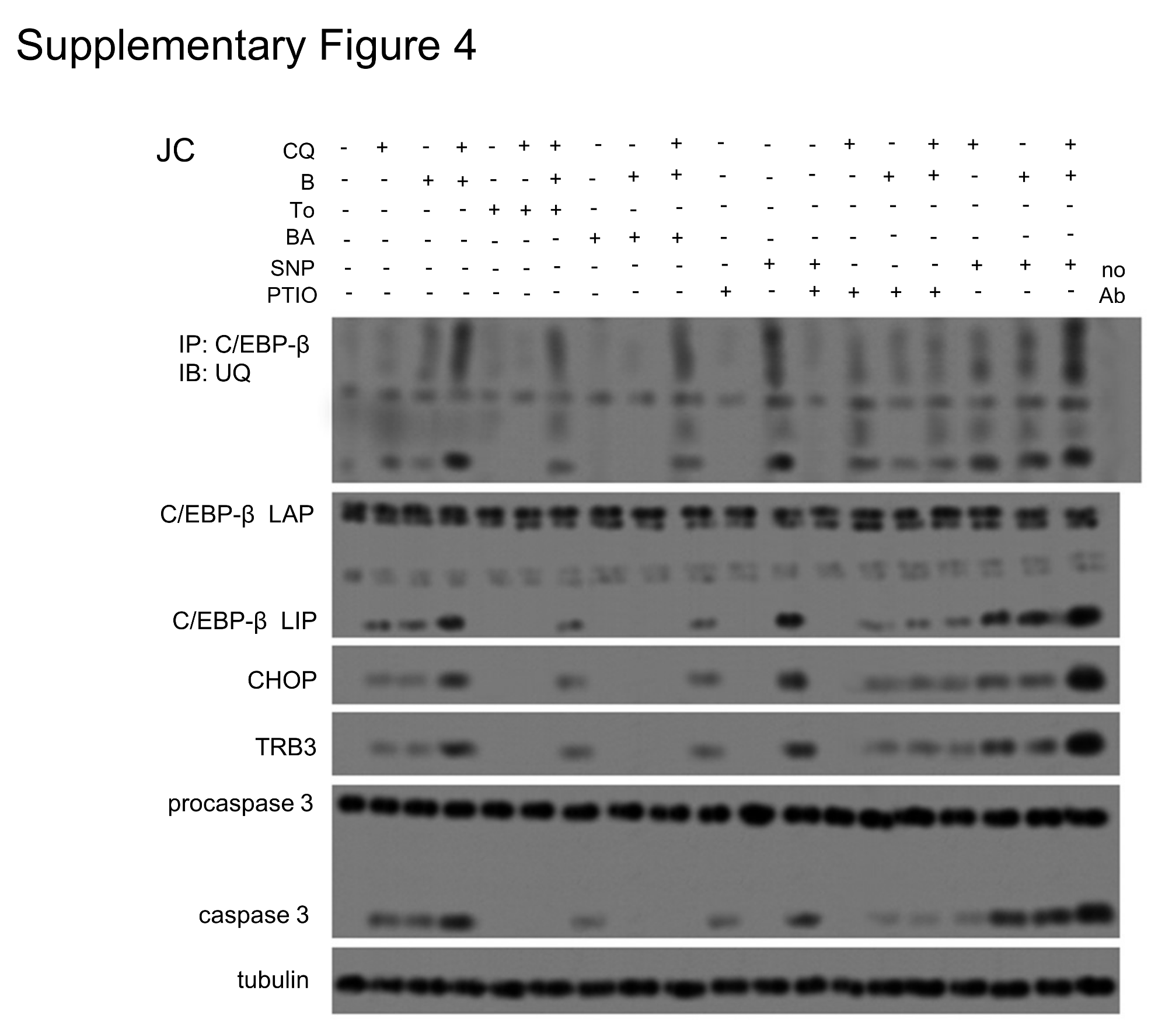
**

**Additional file 5: Figure S4. Effects of lysosome and proteasome inhibitors or activators, NO donor or scavenger on C/EBP-β-LIP and downstream effectors in JC cells**

JC cells were cultured for 24 h in the absence (-) or presence (+) of the lysosome inhibitor chloroquine (CQ; 1 μM), the proteasome inhibitor bortezomib (B; 1 μM), the lysosome activator torin-1 (To; 1 μM), the proteasome activator betulinic acid (BA; 10 μM), the NO donor sodium nitroprusside (SNP; 10 μM), the NO scavenger carboxy-PTIO (PTIO; 100 μM), alone or co-incubated in different combinations. Whole cell lysates were immunoprecipitated (IP) with the anti-C/EBP-β antibody, which recognizes both C/EBP-β-LAP and C/EBP-β-LIP, then immunoblotted (IB) with the anti-mono/poly-ubiquitin (UQ) antibody; alternatively, lysates were directly immunoblotted with the indicated antibodies. No Ab: lysate from untreated cells immunoprecipitated in the absence of the anti-C/EBP-β antibody, as control of specificity. The expression of β-tubulin was used as control of equal protein loading before immunoprecipitation. The figure is representative of 1 out of 3 experiments.
