# Supplementary material for: Increasing intratumor C/EBP-β LIP and nitric oxide levels overcome resistance to doxorubicin in triple negative breast cancer
Source: J Exp Clin Cancer Res. 2018 Nov 27;37:286. doi: 10.1186/s13046-018-0967-0 (PMC6258159; doi:10.1186/s13046-018-0967-0)
Supplement: Supplementary file 6 — Figure S5. Chloroquine and bortezomib reversion of doxorubicin resistance in 3D-cultures. (DOCX 3366 kb) [file 13046_2018_967_MOESM6_ESM.docx]

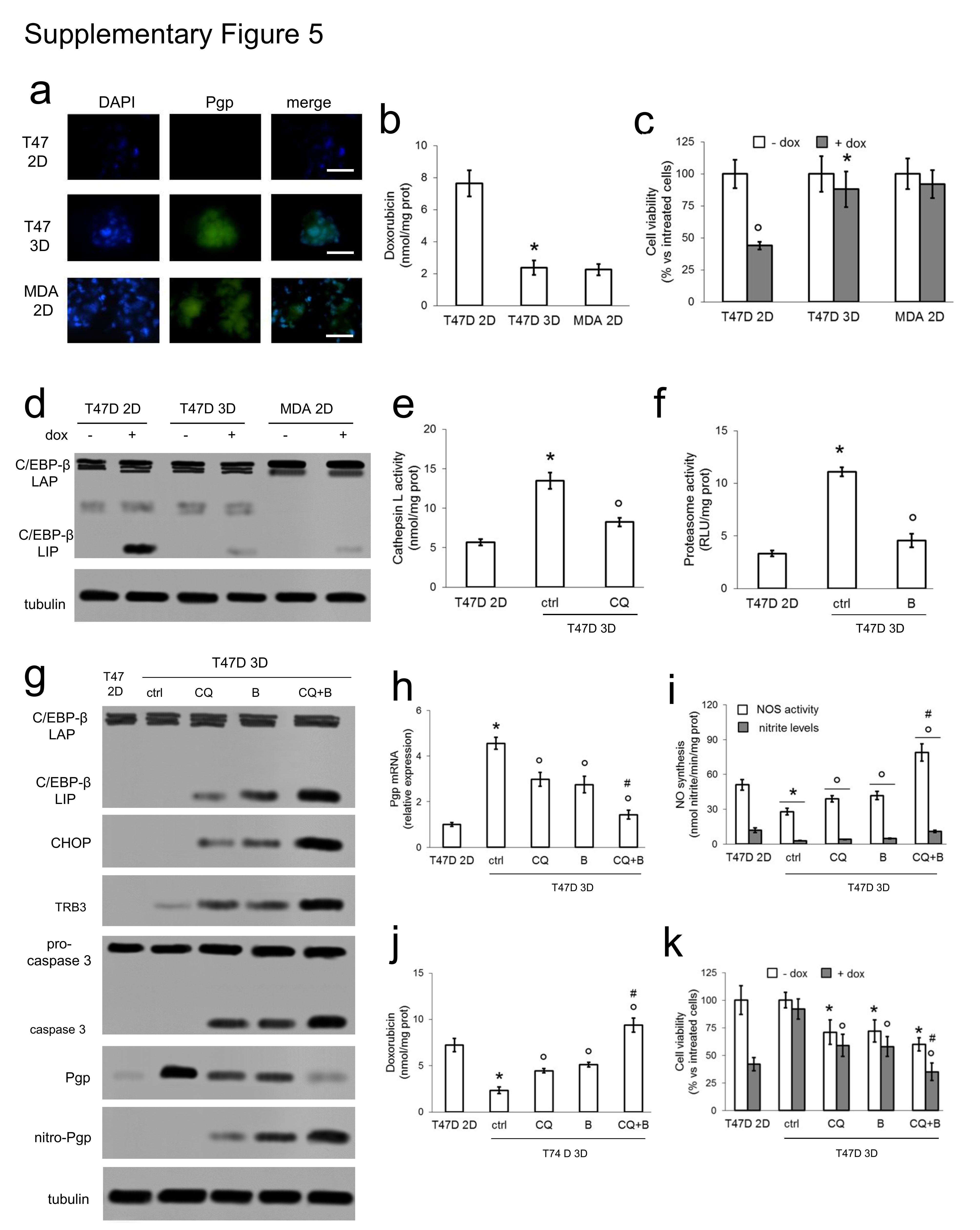


**Additional file 6: Figure S5 Chloroquine and bortezomib reverse doxorubicin resistance in 3D-cultures**

Doxorubicin-sensitive/Pgp-negative T47D cells were growth as bi-dimensional (2D) or tri-dimensional (3D) culture. MDA-MB-231 cells were included as doxorubicin-resistant/Pgp-positive 2D-culture. **a**. Pgp expression in 2D and 3D cultures. Cells were immunostained for Pgp. Nuclei were counter-stained with DAPI (10× ocular lens; 63× objective lens). Bar=100 μm. The photographs are representative of 1 out of 3 experiments. **b.** Cells were cultured in presence of doxorubicin 5 μM for 24h, then the drug accumulation was measured in triplicates by a fluorimetric assay. Data are presented as means±SD (n=3). *p < 0.001: T47D 3D cells vs T47D 2D cells. **c.** Cells were treated with medium without (- dox) or with 5µM doxorubicin (+ dox) for 72 h. Viability was measured in quadruplicates by a chemiluminescence-based assay. Data are presented as means±SD (n=3). *p<0.001: T47D 3D cells vs T47D 2D cells; °p<0.001: “+ dox” cells vs “- dox” cells. **d.** Cells were grown in the absence (-) or presence (+) of 5 µM doxorubicin (dox) for 24h. Whole cell lysates were probed with an antibody recognizing both C/EBP-β LAP and LIP isoforms. The expression of β-tubulin was used as control of equal protein loading. The figure is representative of 1 out of 3 experiments. **e.** Cells were cultured for 24 h in the absence (ctrl) or presence of the lysosome inhibitor chloroquine (CQ; 1 μM). Lysosome activity was analyzed in duplicates by a spectrophotometric assay. Data are presented as means±SD (n=3). *p<0.001: T47D 3D cells vs T47D 2D cells; °p<0.001: CQ-treated T47D 3D cells vs untreated (ctrl) T47D 3D cells. **f.** Cells were cultured for 24 h in the absence (ctrl) or presence of the proteasome inhibitor bortezomib (B; 1 μM). Proteasome activity was analyzed in duplicates by a chemiluminescence-based assay. Data are presented as means±SD (n=3). *p<0.001: T47D 3D cells vs T47D 2D cells; °p<0.001: CQ-treated T47D 3D cells vs untreated (ctrl) T47D 3D cells. **g.** T47D 3D cells were cultured 24 h in the absence (ctrl) or presence of the lysosome inhibitor chloroquine (CQ; 1 μM) or the proteasome inhibitor bortezomib (B; 1 μM), alone or in combination. Whole cell lysates were probed with the indicated antibodies. To detect nitrated Pgp (nitroPgp), extracts were immunoprecipitated with an antibody anti-nitrotyrosine, then probed for Pgp. The expression of β-tubulin was used as control of equal protein loading. The figure is representative of 1 out of 3 experiments. **h.** The relative expression of *Pgp* gene was measured by qRT-PCR in cells treated as in **g**. Data are presented as mean±SD (n=4). *p<0.001: T47D 3D cells vs T47D 2D cells; °p<0.001: CQ/B/CQ+B-treated 3D cells vs untreated (ctrl) 3D cells; ^#^p<0.001: CQ+B-treated 3D cells vs CQ/B-treated 3D cells. **i.** The activity of NOS enzymes in cell lysate or nitrite levels in the supernatant were measured in triplicates by spectrophotometric assays. Data are presented as mean±SD (n=3). *p<0.001: T74D 3D cells vs T47D 2D cells; °p<0.02: CQ/B/CQ+B-treated 3D cells vs untreated (ctrl) 3D cells; ^#^p<0.001: CQ+B-treated 3D cells vs CQ/B-treated 3D cells. **j.** Cells were cultured as indicated in **g**, followed by 5 μM doxorubicin for 24h further. Drug accumulation was measured in triplicates by a fluorimetric assays. Data are presented as means±SD (n=3). *p<0.001: T47D 3D cells vs T47D 2D cells; °p<0.001: CQ/B/CQ+B-treated 3D cells vs untreated (ctrl) 3D cells; ^#^p<0.001: CQ+B-treated 3D cells vs CQ/B-treated 3D cells. **k.** Cells, incubated for 24 h as reported in **g**, were treated with (+) or without (- dox) or with (+ dox) 5µM doxorubicin for 48 h further. Viability was measured in quadruplicates by a chemiluminescence-based assay Data are presented as means±SD (n=3). *p<0.05: T47D 3D cells vs T47D 2D cells; °p<0.01: CQ/B/CQ+B-treated 3D cells vs untreated (ctrl) 3D cells; ^#^p<0.05: CQ+B-treated 3D cells vs CQ/B-treated 3D cells.
