# Supplementary material for: Increasing intratumor C/EBP-β LIP and nitric oxide levels overcome resistance to doxorubicin in triple negative breast cancer
Source: J Exp Clin Cancer Res. 2018 Nov 27;37:286. doi: 10.1186/s13046-018-0967-0 (PMC6258159; doi:10.1186/s13046-018-0967-0)
Supplement: Supplementary file 7 — Figure S6. Set up and validation of an inducible C/EBP-β LIP expression system in Pgp-positive/doxorubicin-resistant JC cells. (DOCX 1224 kb) [file 13046_2018_967_MOESM7_ESM.docx]

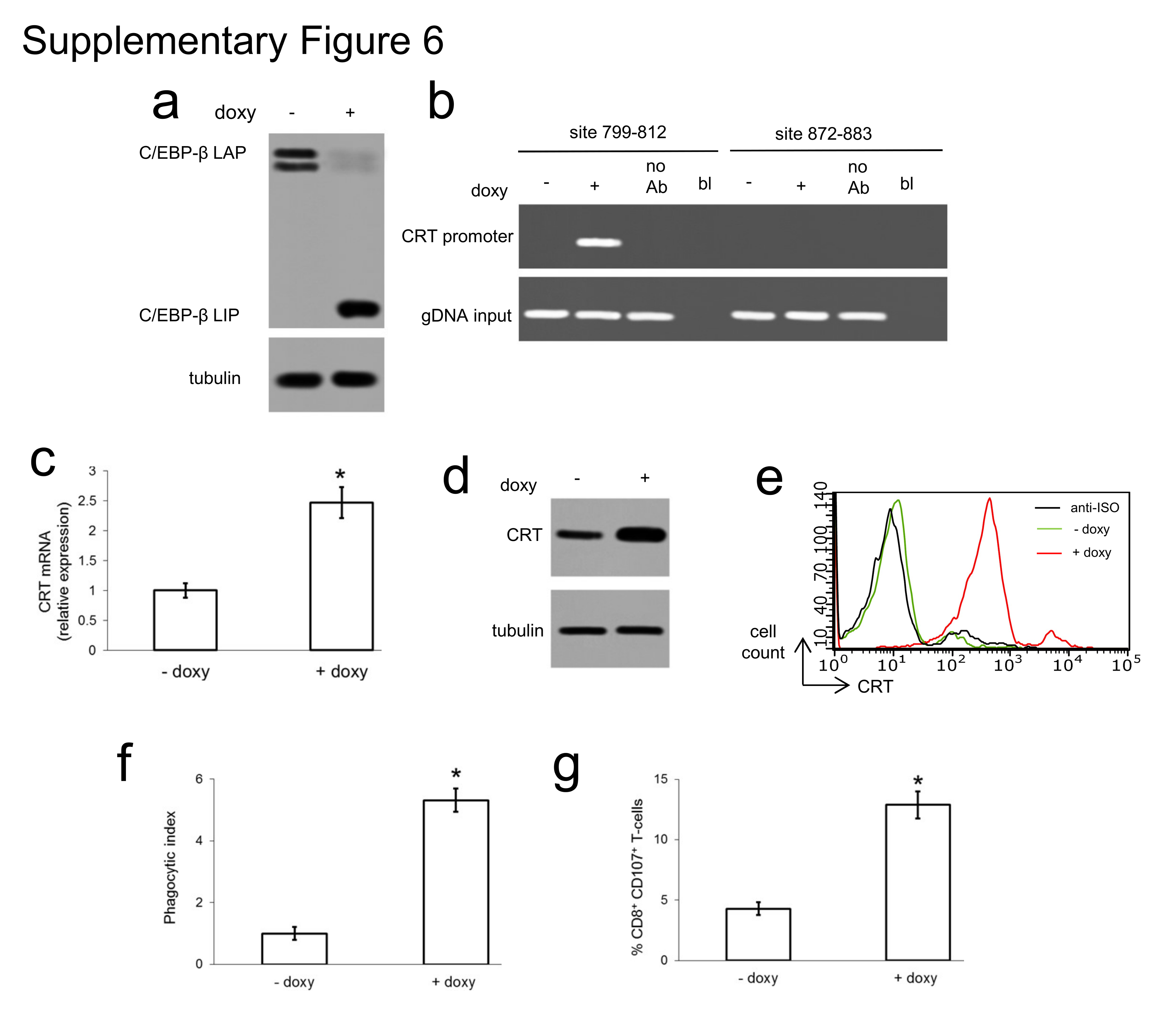


**Additional file 7: Figure S6. Set up and validation of an inducible C/EBP-β LIP expression system in Pgp-positive/doxorubicin-resistant JC cells**

JC cells were stably transfected with a doxycycline-inducible (TetON) vector encoding C/EBP-β LIP. Cells were cultured in the absence (-) or presence (+) of doxycycline (doxy; 1 μg/ml) for 24 h, to induce C/EBP-β LIP. **a.** Whole cell lysates were probed with an antibody recognizing both C/EBP-β LAP and LIP isoforms. The expression of β-tubulin was used as control of equal protein loading. The figure is representative of 1 out of 3 experiments. **b.** ChIP was performed to evaluate the binding of LIP to *CRT* promoter (sites: 799-812; 872-883). no Ab: no anti-C/EBP-β antibody; bl: blank; DNA input: genomic DNA. The figure is representative of 1 out of 3 experiments. **c.** The relative expression of *CRT* gene was measured in triplicates by qRT-PCR. Data are presented as means±SD (n=3). *p<0.001: “+ doxy” vs “- doxy” cells. **d.** Whole cell lysates were probed with an anti-CRT antibody. The expression of β-tubulin was used as control of equal protein loading. The figure is representative of 1 out of 3 experiments. **e.** Surface CRT was detected by flow cytometry . The histograms represent the results obtained from 1 out of 3 experiments. Anti-ISO: anti-isotype antibody. **f.** Tumor cells were stained with PKH2-FITC, DC were stained with an anti-HLA-DR-PE antibody. Tumor cells were co-incubated with DC for 24 h. Double-stained cells were counted by flow cytometry. Phagocytic index was considered an index of phagocytosis rate. Data are presented as means±SD (n=3). *p<0.001: “+ doxy” vs “- doxy” cells. **g.** T-lymphocytes were co-cultured with DC after phagocytosis, then incubated with JC cells. The percentage of CD8^+^CD107^+^T-cells was measured by flow cytometry. Data are presented as means±SD (n=3). *p<0.001: “+ doxy” vs “- doxy” cells.
