# Supplementary material for: Increasing intratumor C/EBP-β LIP and nitric oxide levels overcome resistance to doxorubicin in triple negative breast cancer
Source: J Exp Clin Cancer Res. 2018 Nov 27;37:286. doi: 10.1186/s13046-018-0967-0 (PMC6258159; doi:10.1186/s13046-018-0967-0)
Supplement: Supplementary file 8 — Figure S7. C/EBP-β LIP levels in TetON MDA-MB-231 cells, treated with chloroquine, bortezomib and doxorubicin. (DOCX 1723 kb) [file 13046_2018_967_MOESM8_ESM.docx]

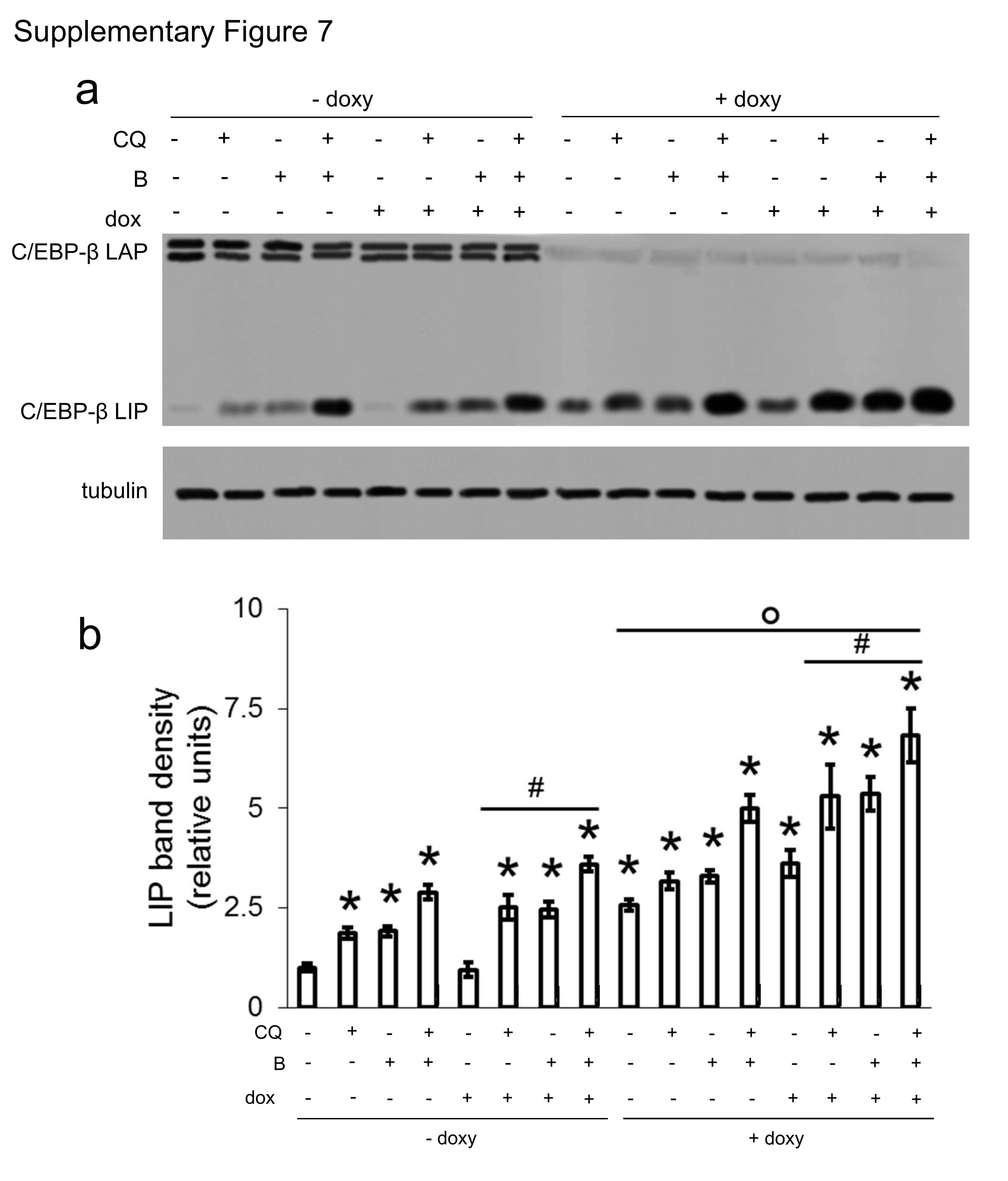


**Additional file 8: Figure S7. C/EBP-β LIP levels in TetON MDA-MB-231 cells, treated with chloroquine, bortezomib and doxorubicin**

MDA-MB-231 cells were stably transfected with a doxycycline-inducible vector encoding C/EBP-β LIP, cultured in the absence (-) or presence (+) of doxycycline (doxy; 1 μg/ml) for 24 h to induce C/EBP-β LIP. When indicated, cells were co-incubated with the lysosome inhibitor chloroquine (CQ; 1 μM) or with the proteasome inhibitor bortezomib (B; 1 μM), alone or in combination, followed by 5 μM doxorubicin (dox) for further 24 h. **a.** Whole cell lysates were probed with an antibody recognizing both C/EBP-β LAP and LIP isoforms. The expression of β-tubulin was used as control of equal protein loading. The figure is representative of 1 out of 3 experiments. **b.** The densitometric analysis of LIP was expressed as mean band density±SD, where the mean density in untreated cells was expressed as 1. *p<0.001: all treatments vs “- doxy, ctrl” group; °p<0.005: “+ doxy” treatments vs. corresponding “- doxy” treatments; ^#^p<0.01: “+ dox” treatments vs. corresponding “- dox” treatments.
