# Supplementary material for: Increasing intratumor C/EBP-β LIP and nitric oxide levels overcome resistance to doxorubicin in triple negative breast cancer
Source: J Exp Clin Cancer Res. 2018 Nov 27;37:286. doi: 10.1186/s13046-018-0967-0 (PMC6258159; doi:10.1186/s13046-018-0967-0)
Supplement: Supplementary file 9 — Figure S8. Effects of CHOP silencing on nitric oxide production, Pgp expression and activity, calreticulin expression. (DOCX 3230 kb) [file 13046_2018_967_MOESM9_ESM.docx]

**
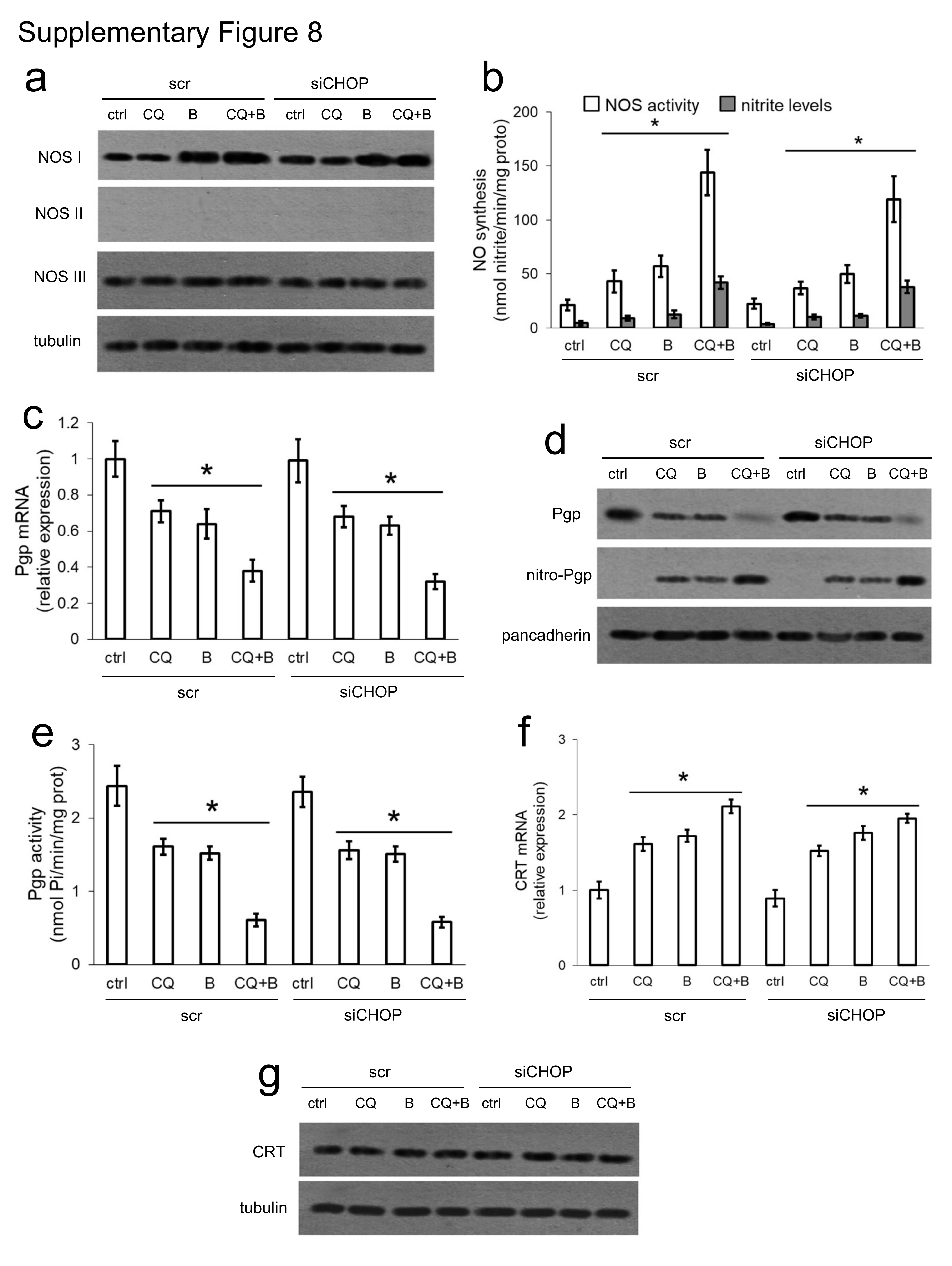
**

**Additional file 9: Figure S8. Effects of CHOP silencing on nitric oxide production, Pgp expression and activity, calreticulin expression**

MDA-MB-231 cells were transfected with a non-targeting siRNA (scrambled; scr) or with a CHOP-targeting siRNAs pool (siCHOP). Cells were grown in fresh medium (ctrl) or in a medium containing the lysosome inhibitor chloroquine (CQ; 1 μM) or the proteasome inhibitor bortezomib (B; 1 μM), alone or in combination, for 24 h further. **a.** Whole cell lysates were probed for NOS I, NOS II, NOS III. The expression of β-tubulin was used as control of equal protein loading. The figure is representative of 1 out of 3 experiments. **b.** The activity of NOS enzyme in cell lysate and the levels of nitrite in the supernatants were measured in triplicates by spectrophotometric assays. Data are mean±SD (n=3). *p<0.05: CQ/B/CQ+B-treated cells vs “scr ctrl” cells. **c.** The relative expression of *Pgp* gene was measured by qRT-PCR. Data are presented as mean±SD (n=3). *p<0.005: CQ/B/CQ+B-treated cells vs “scr ctrl” cells. **d.** Plasma-membrane extracts were probed for Pgp or immuno-precipitated with an anti-nitrotyrosine antibody, then probed for Pgp (nitroPgp). The expression of pancadherin was used as control of equal membrane protein loading. The figure is representative of 1 out of 3 experiments. **e.** Pgp activity was analyzed in duplicates by a spectrophotometric assay. Data are presented as mean±SD (n=3). *p<0.001: CQ/B/CQ+B-treated cells vs “scr ctrl” cells. **f.** The relative expression of *CRT* gene were measured in triplicates by qRT-PCR. Data are presented as means±SD (n=3). * p<0.005: CQ/B/CQ+B-treated cells vs “scr ctrl” cells. **g.** Whole cell lysates were probed with an anti-CRT antibody. The expression of β-tubulin was used as control of equal protein loading. The figure is representative of 1 out of 3 experiments.
