# Supplementary material for: Increasing intratumor C/EBP-β LIP and nitric oxide levels overcome resistance to doxorubicin in triple negative breast cancer
Source: J Exp Clin Cancer Res. 2018 Nov 27;37:286. doi: 10.1186/s13046-018-0967-0 (PMC6258159; doi:10.1186/s13046-018-0967-0)
Supplement: Supplementary file 10 — Figure S9. Immunohistochemical and immunological parameters of mice exposed to chloroquine, bortezomib and doxorubicin. (DOCX 2475 kb) [file 13046_2018_967_MOESM10_ESM.docx]

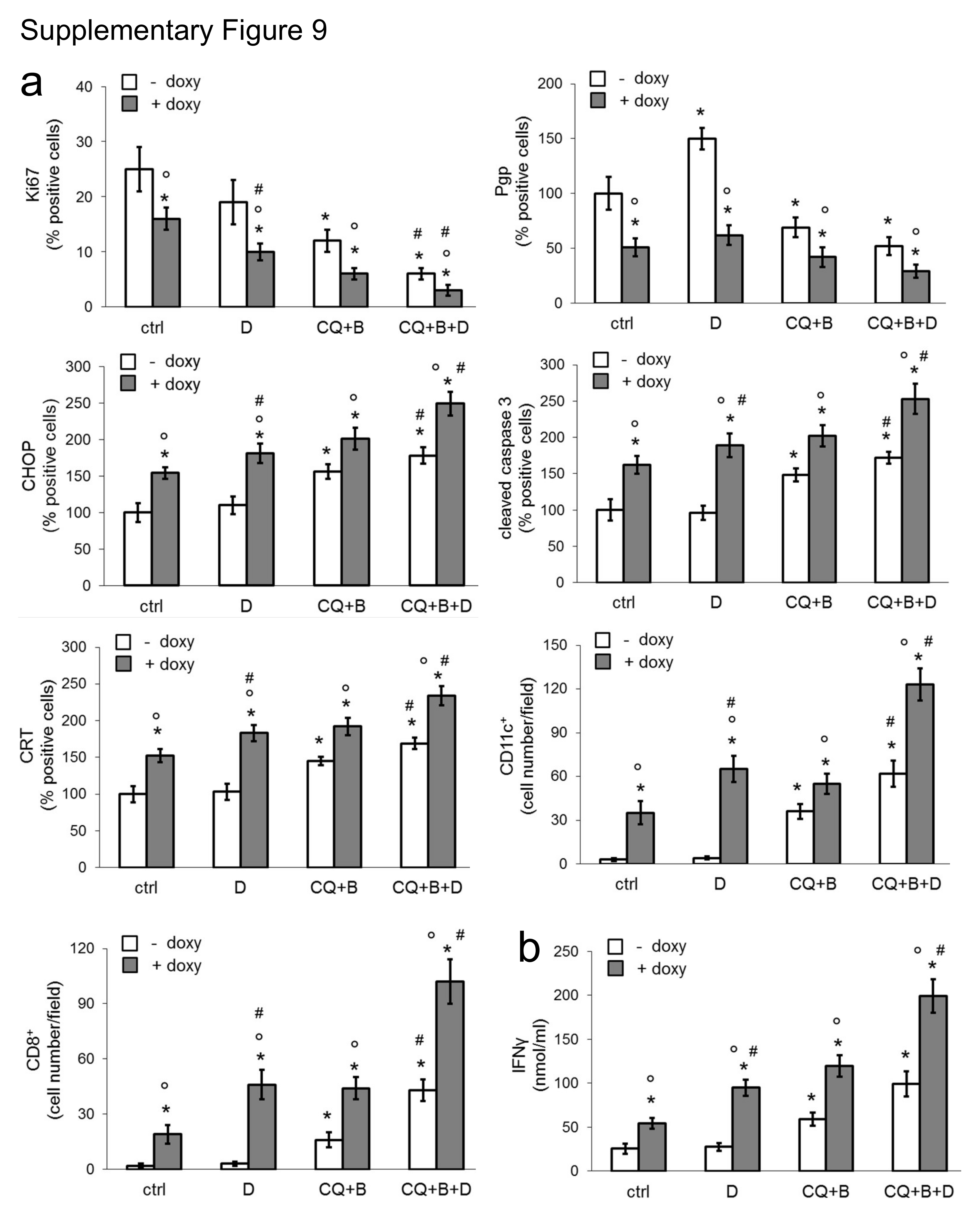


**Additional file 10: Figure S9. Immunohistochemical and immunological parameters of tumors from mice exposed to chloroquine, bortezomib and doxorubicin**

Quantification of immunostaining of Figure 8**c**. The percentage of proliferating cells was determined by the ratio of Ki67-positive nuclei and the total cell count (hematoxylin-positive nuclei; 108-99 nuclei/field). The percentage of Pgp, CHOP, cleaved caspase 3 and CRT-positive cells was determined by analyzing the sections of each group (105-93 cells/field): “ctrl” group intensity was considered as 100%. The number of CD11c^+^ and CD8^+^ cells/field was counted on 5 field/experimental conditions. The analyses were performed with the ImageJ software. *p<0.01: all treatments vs. “- doxy, ctrl” group; °p<0.02: “+ doxy” treatments vs. corresponding “- doxy” treatments; ^#^p<0.02: “+ dox” treatments vs. corresponding “- dox” treatments. **b**. IFN-γ was measured in duplicates by ELISA in the supernatants of tumor-draining lymph nodes. Data are presented as means±SD. *p<0.001: all treatments vs. “- doxy, ctrl” group; ° p<0.005: “+ doxy” treatments vs. corresponding “- doxy” treatments; ^#^p<0.01: “+dox” treatments vs. corresponding “-dox” treatments.
