# Supplementary material for: Increasing intratumor C/EBP-β LIP and nitric oxide levels overcome resistance to doxorubicin in triple negative breast cancer
Source: J Exp Clin Cancer Res. 2018 Nov 27;37:286. doi: 10.1186/s13046-018-0967-0 (PMC6258159; doi:10.1186/s13046-018-0967-0)
Supplement: Supplementary file 11 — Table S2 Hematochemical parameters of animals treated with doxorubicin, chloroquine and bortezomib, in the presence of intratumorally induced C/EBP-β LIP. (DOCX 16 kb) [file 13046_2018_967_MOESM11_ESM.docx]

**Additional file 11: Table S2 Hematochemical parameters of animals treated with doxorubicin, chloroquine and bortezomib, in the presence of intratumorally induced C/EBP-β LIP**

| **- doxy** | ctrl | dox | C+B | C+B+D |
| --- | --- | --- | --- | --- |
| LDH (U/L) | 6073 + 837 | 6278 + 389 | 6118 + 672 | 6342 + 715 |
| AST (U/L) | 109 + 35 | 134 + 73 | 127 + 23 | 142 + 102 |
| ALT (U/L) | 32 + 4 | 38 + 6 | 33 + 9 | 39 + 12 |
| AP (U/L) | 102 + 27 | 127 + 33 | 109 + 11 | 132 + 14 |
| Creatinine (mg/L) | 0.065 + 0.004 | 0.078 + 0.009 | 0.074 + 0.012 | 0.072 + 0.007 |
| CPK (U/L) | 287 + 82 | 661 + 102 * | 271 + 39 | 672 + 94 * |
| CPK-MB (ng/mL) | 0.123 + 0.044 | 0.309 + 0.029 * | 0.142 + 0.072 | 0.289 + 0.017 * |
| cTnI (pg/mL) | 1.034 + 0.071 | 1.009 + 0.045 | 1.002 + 0.052 | 1.052 + 0.033 |
| cTnT (pg/mL) | 2.085 + 0.412 | 2.912 + 0.071 * | 1.983 + 0.054 | 2.872 + 0.111 * |

| **+ doxy** | ctrl | dox | C+B | C+B+D |
| --- | --- | --- | --- | --- |
| LDH (U/L) | 5783 + 528 | 6029 + 442 | 6720 + 639 | 6891 + 872 |
| AST (U/L) | 94 + 21 | 127 + 62 | 91 + 30 | 103 + 62 |
| ALT (U/L) | 38 + 11 | 43 + 12 | 33 + 4 | 42 + 10 |
| AP (U/L) | 128 + 44 | 136 + 67 | 129 + 39 | 162 + 43 |
| Creatinine (mg/L) | 0.073 + 0.007 | 0.072 + 0.004 | 0.063 + 0.008 | 0.071 + 0.009 |
| CPK (U/L) | 234 + 34 | 546 + 84 * | 243 + 72 | 602 + 88 * |
| CPK-MB (ng/mL) | 0.117 + 0.056 | 0.294 + 0.033 * | 0.123 + 0.082 | 0.324 + 0.014 * |
| cTnI (pg/mL) | 1.023 + 0.053 | 1.028 + 0.067 | 1.029 + 0.043 | 1.032 + 0.062 |
| cTnT (pg/mL) | 1.973 + 0.309 | 3.067 + 0.172 * | 2.003 + 0.077 | 2.993 + 0.183 * |

Balb/C mice (n=10 animals/group) were treated as described in Figure **8a**. Blood was collected immediately after euthanasia and analyzed for lactate dehydrogenase (LDH), aspartate aminotransferase (AST), alanine aminotransferase (ALT), alkaline phosphatase (AP), creatinine, creatine phosphokinase (CPK) and CPK-MB, cardiac troponin I (cTnI) and T (cTnT). -doxy/+doxy: absence or presence of 1 mg/ml doxycycline in the drinking water. Ctrl: control group; dox: doxorubicin-receiving group; C+B: chloroquine+bortezomib-receiving group; C+B+D: chloroquine +bortezomib+doxorubicin-receiving group. Data are presented as means±SD. * p < 0.05: vs ctrl group.
